# *Helicobacter pylori* induces the production of interleukin-37 to promote broad immunosuppression and enhance colonization

**DOI:** 10.1080/19490976.2026.2618860

**Published:** 2026-02-14

**Authors:** Rishi Pathirana, Nagaja Capitani, Christopher McCrory, Chiara Della Bella, Isabella Stuart, William Gilmore, Natalie J. Bitto, Michelle D. Tate, Ella L. Johnston, Lauren Zavan, Steven Batinovic, John Pedersen, Sam Norden, Hassan Chaudhry, Richard L. Ferrero, Tony M. Korman, David Greening, Neil O’Brien-Simpson, Andrew M. Ellisdon, James C. Whisstock, Steve Petrovski, Renea A. Taylor, Mario M. D’Elios, Claudia A. Nold-Petry, Marcel F. Nold, Maria Kaparakis-Liaskos

**Affiliations:** a Department of Microbiology, Anatomy, Physiology and Pharmacology, La Trobe University, Bundoora, Victoria, Australia; b Department of Life Sciences, University of Siena, Siena, Italy; c Department of Microbiology and Immunology, The Peter Doherty Institute for Infection and Immunity, University of Melbourne, Victoria, Australia; d Department of Molecular and Developmental Medicine, University of Siena, Siena, Italy; e Centre for Innate Immunity and Infectious Diseases, Hudson Institute of Medical Research, Clayton, Victoria, Australia; f Department of Molecular and Translational Sciences, Monash University, Melbourne, Victoria, Australia; g TissuPath Specialist Pathologists, Mount Waverley, Victoria, Australia; h Biomedicine Discovery Institute, Department of Microbiology, Monash University, Melbourne, Victoria, Australia; i Centre for Inflammatory Diseases, Monash University, Melbourne, Victoria, Australia; j Molecular Proteomics, Baker Heart and Diabetes Institute, Victoria, Australia; k Baker Department of Cardiovascular Research, Translation and Implementation, La Trobe University, Victoria, Australia; l ACTV Research Group, Division of Basic and Clinical Oral Sciences, Melbourne Dental, School, Royal Dental Hospital, The University of Melbourne, Carlton, Victoria, Australia; m Department of Biochemistry and Molecular Biology, Biomedicine Discovery Institute, Monash University, Melbourne, Victoria, Australia; n Department of Physiology, Biomedicine Discovery Institute, Monash University, Melbourne, Victoria, Australia; o Department of Oncology, Peter MacCallum Cancer Centre, The University of Melbourne, Victoria, Australia; p Department of Paediatrics, Monash University, Melbourne, Victoria, Australia; q Ritchie Centre, Hudson Institute of Medical Research, Melbourne, Victoria, Australia; r Monash Newborn, Monash Children’s Hospital, Melbourne, Victoria, Australia

**Keywords:** *Helicobacter pylori*, interleukin-37 (IL-37), bacterial pathogenesis, NOD1, TLRs, innate immunity, adaptive immunity, immune suppression, gastric inflammation, T cells

## Abstract

*Helicobacter pylori* infects approximately half of the world’s population, resulting in lifelong gastric infections. To promote lifelong colonization, *H.* *pylori* modulates host immunity via unknown mechanisms. Here we show that *H.* *pylori* can harness the pan-immunosuppressive cytokine interleukin-37 (IL-37) to facilitate pathogenesis, enhance colonization and prevent the development of innate, cellular and humoral immunity. We show that *H.* *pylori* increased the production of IL-37 in human gastric biopsies, and IL-37 secretion by gastric epithelial cells and human gastric mucosoids. We found that *H.* *pylori* induced IL-37 secretion by epithelial cells via activation of host pathogen recognition receptors TLR2, TLR4 and NOD1, in addition to the *H.* *pylori*-encoded cag pathogenicity island, revealing that *H.* *pylori* utilises multiple mechanisms to induce IL-37 production during infection. Once produced, IL-37 attenuated TLR2, TLR4 and NOD1-mediated activation and TLR-mediated IL-8 responses to *H.* *pylori* infection. Using transgenic mice expressing human IL-37, we found that IL-37 promoted immunosuppression by significantly increasing *H.* *pylori* colonization, limiting gastric inflammation, and reducing *H.* *pylori*-specific antibody responses. Furthermore, we identified the multiple mechanisms whereby IL-37 functions to impair the development of adaptive immunity. IL-37 abolished human T and B cell responses by impairing their activation, migration, and preventing immune synapse formation. Moreover, IL-37 inhibited proliferation of gastric-derived *H.* *pylori*-specific T cells isolated from *H.* *pylori*-infected patients, revealing a mechanism whereby IL-37 functions to prevent pathogen-specific cellular immune responses. Collectively, our findings reveal that *H.* *pylori* induces production of the pan-immunosuppressive cytokine IL-37 to enhance colonization, modulate gastritis and suppress innate, cellular and humoral immunity to ultimately promote pathogenesis in the host. These findings advance our understanding of *H.* *pylori*-mediated disease and identify gastric IL-37 as a therapeutic target to combat *H.* *pylori* infection and associated diseases.

## Graphical Abstract

**Figure:**
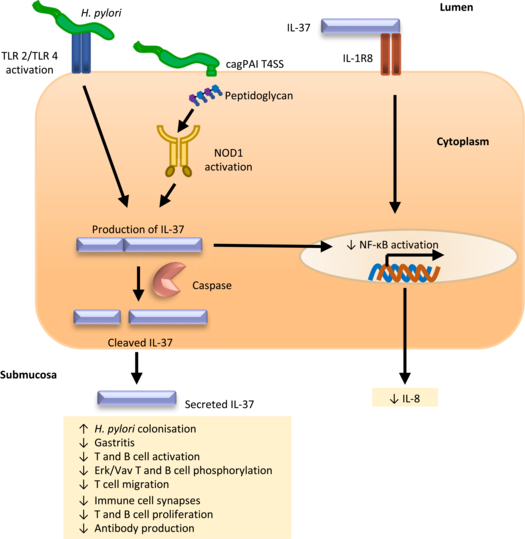

## Introduction

*Helicobacter pylori* infects the stomach of more than 3 billion people worldwide and persists within the host for life due to its ability to modulate the host’s immune response.^1^^-^^3^ Infection with *H.* *pylori* commonly occurs during early childhood and results in a spectrum of diseases ranging from gastritis in all infected individuals, to gastric ulcers or cancer, with approximately 75% of gastric cancers attributable to *H.* *pylori* infection.^1^^-^^3^ It is well established that *H.* *pylori* manipulates the host’s immune system into mounting an ineffective yet chronic immune response, and it is suggested that this pathogen-mediated immunomodulation facilitates persistence and disease progression.^2^^,^^4-6^ Some of the mechanisms used by *H.* *pylori* to modulate immunity involve modifications in LPS to avoid detection by TLR4^7^, in addition to inhibition of T and B cell proliferation and dendritic cell activation by the vacuolating toxin (VacA)^8^^,^^9^ and the γ-glutamyl transpeptidase.^10^^,^^11^ However, further examination of the underlying mechanisms utilized by *H.* *pylori* to manipulate host immunity and promote immunomodulation will provide greater insights into the mechanisms used by *H.* *pylori* to establish lifelong colonization and will identify novel therapeutic targets to treat *H.* *pylori* infections.

We previously reported that interleukin-37 (IL-37) functions as a fundamental inhibitor of innate and adaptive immunity.^12^^-^^14^ IL-37 is expressed by many host cells in response to activation of innate immune pattern recognition receptors (PRRs), such as Toll-like receptor 4 (TLR4) activation in response to bacterial lipopolysaccharide (LPS).^12^ Once produced, IL-37 substantially reduces the production of pro-inflammatory cytokines and attenuates innate immunity, thus functioning as a potent anti-inflammatory agent that prevents excessive inflammation triggered via TLR activation.^12^^,^^15^ As *H.* *pylori* can activate a range of host PRRs, we speculated that PRR activation by *H.* *pylori* could result in the production of IL-37, which could subsequently function to promote immunomodulation, bacterial colonization and chronic disease progression in individuals.

Here we show that IL-37, a powerful pan-immunosuppressive and anti-inflammatory IL-1 family cytokine, facilitates *H.* *pylori*-mediated broad immunomodulation. We found that *H.* *pylori* induced the production of IL-37 in human gastric tissue and gastric mucosoids, and the secretion of IL-37 by human gastric epithelial cells in a TLR2, TLR4 and NOD1-dependent manner. Furthermore, we identified that the presence of the cag pathogenicity island (cagPAI) and cagA in *H.* *pylori* strains enhanced IL-37 production by host cells, and that secretion of IL-37 by gastric epithelial cells relied on caspase and inflammasome activity. Using transgenic mice expressing human IL-37, we found that IL-37 enhanced *H.* *pylori* colonization while impairing immune cell recruitment into the gastric tissue and prevented the development of *H.* *pylori*-specific antibody responses. Furthermore, we elucidated the multiple mechanisms whereby IL-37 functions to abolish human immune cell functions to ultimately promote immunosuppression and prevent the development of an effective adaptive immune response. Collectively, these findings reveal that *H.* *pylori* harnesses the powerful pan-immunosuppressive functions of IL-37 to suppress host immunity and promote colonization, and thus identifies IL-37 blockade as a novel therapeutic strategy to treat chronic *H.* *pylori* infections.

## Materials and methods

### Immunohistochemistry of human gastric biopsies

Human Research Ethics Committee approval (Project ID 20623) was obtained from Monash University Human ethics committee to allow access to retrospectively archived formalin fixed paraffin embedded tissues. A comparative cohort of patients with *H.* *pylori* or non-infected controls were recruited for pathology review. Haematoxylin and Eosin-stained sections from fresh-frozen or formalin-fixed paraffin-embedded (FFPE) stomach tissues from infected and non-infected individuals were assessed by clinical pathologists (J.P., S.N.). Regions of at least 70% epithelial cellularity were manually micro-dissected from 5 μm FFPE sections. Tissues were processed for 12 h using the fat processing run on the Leica PELORIS rapid tissue processer (Leica Biosystems), and stained with Haematoxylin (Thermo Fisher Scientific) and Eosin (Amber scientific, 1% aqueous eosin) using a Leica- RST autostainer (Leica Biosystems). For immunohistochemistry, tissues were stained using a Ventana Benchmark Ultra, using Ventana/Roche reagents and kits (Roche). Antigen retrieval was performed with acidic citrate buffer, pH 6 for 24 min using a Ventana/Roche CC2. Tissues were incubated with primary antibody (anti-IL-37 antibody, 1:500 dilution, eBioscience, clone 37D12) for 20 min at 36 °C then detected using a Ventana/Roche Optiview DAB detection kit (Roche).

### qRT-PCR of human gastric biopsies

Total RNA from formalin-fixed, paraffin-embedded tissue sections was isolated using RNeasy FFPE Kit (Qiagen) with an on-column DNase treatment according to manufacturer’s protocol. RNA concentrations were quantified using NanoDrop™ 2000 spectrophotometer (Thermo Fisher Scientific). Reverse transcription was performed with 1 μg RNA using the iScript gDNA clear cDNA synthesis kit (BioRad). The cDNA was pre-amplified using the pre-amplification Master Mix (Thermo Fisher Scientific) according to the manufacturer’s instructions. For gene expression using qRT-PCR, a TaqMan Gene Expression assay Master Mix was used with the following TaqMan primer-probe set for *IL37* (Hs00367201_m1, Ambion) as per manufacturer’s instructions. All qRT-PCR results were normalized using *18S* (TaqMan Assay ID: Hs99999901_s1), and reactions were performed using a Touch Real-Time CFX96 PCR detection system (BioRad). The fold change in gene expression levels relative to 18 s was determined by the 2^(-Delta Delta Cq) method.

### Bacterial strains and culture conditions

*H.* *pylori* 251,^16^ 251 Δ*cag*PAI isogenic mutant,^16^ 251 Δ*cag*A isogenic mutant,^17^ the *H.* *pylori* clinical isolates MMC2, MMC6, MMC10, MMC17, MMC19 (this study), and the mouse adapted *H.* *pylori* Sydney Strain 1 (SS1),^16^ were grown on Horse blood agar (HBA) comprised of blood agar base No. 2 (Oxoid), supplemented with 5% (v/v) horse blood, and Skirrow’s selective supplement (155 μg/ml Polymyxin B, 6.25 μg/ml vancomycin, 3.12 μg/ml trimethoprim, and 1.25 mg/ml amphotericin B, all from Sigma-Aldrich). All *H.* *pylori* strains were cultured at 37 °C using microaerophilic conditions (Campygen, Oxoid).^16^

For use in cell co-culture assays and mouse infections, *H.* *pylori* strains were grown in brain heart infusion broth (Oxoid) supplemented with 10% (v/v) heat-inactivated newborn calf serum (Gibco) and Skirrow’s selective media and were incubated overnight at 37 °C with shaking at 120 rpm and using microaerophilic conditions.^16^

*H.* *pylori* grown in liquid cultures were pelleted, washed with PBS and resuspended in plain cell culture media for use in cell stimulation assays, or in PBS for mouse colonization experiments.^16^ The number of *H.* *pylori* used to stimulate cells or infect mice was determined by performing serial dilutions and enumerating colony forming units on HBA.

### Illumina sequencing of *H.* *pylori* isolates

For Illumina sequencing, genomic DNA (50 ng) was prepared from all *H.* *pylori* isolates using the NEBNext® Ultra™ II FS DNA Library Prep Kit (NEB). The DNA was whole genome sequenced on an Illumina MiSeq v3 600-cycle kit (Illumina). The DNA sequence was quality trimmed using Trim Galore and assembled using Unicycler.^18^

### Cell culture

Human adenocarcinoma gastric epithelial (AGS) cells and Jurkat T cells were grown in RPMI 1640 (Gibco, USA), and HEK293 cells were grown in DMEM (Gibco, USA). All media was supplemented with 10% (v/v) FCS, 1% (v/v) L-glutamine and 1% (v/v) penicillin/streptomycin (Gibco, USA). TLR2, TLR4 and NOD1 HEK Blue™ reporter cells (InvivoGen, USA) were cultured in DMEM supplemented with 10% (v/v) FCS, 1% (v/v) L-glutamine and 1% (v/v) penicillin/streptomycin (Gibco, USA) with selective antibiotics for each cell line: Zeocin (100 μg/mL) and Blasticidin (30 μg/mL) for TLR2, TLR4 and NOD1 HEK-Blue cells, and Hygromycin (200 μg/mL) for TLR2 and TLR4 HEK-Blue cells (InvivoGen).^19^^-^^21^ All cells were grown at 37 °C with 5% CO_2_.

### *H.* *pylori* cell co-culture assays

AGS, HEK293 and HEK-Blue reporter (InvivoGen) cells were seeded in 24 well cell culture plates (1 × 10^5^ cells/well), grown overnight at 37 °C in 5% CO_2_, and used the following day for transfection, stimulation or Western blot analyses.

For *H.* *pylori* mediated IL-37 secretion and HEK-Blue activation studies, AGS and HEK-Blue cells were transfected the following day with 300 ng/well of a pIRES vector expressing IL-37b^12^ or 300 ng/well of control pIRES vector lacking the IL-37b insert (control). AGS cells were also co-transfected with 300 ng/well of TLR2 or TLR4-MD-2 expression constructs (Gift from Ashley Mansell),^22^ or 50 ng of NOD1.^16^^,^^23^ The total DNA used to transfect AGS or HEK-Blue cells was adjusted to 1150 ng per well using pcDNA_3_, and cells were transfected using Lipofectamine 2000 reagent (Invitrogen), as previously described.^16^^,^^23^ The following day, culture media was replaced with plain culture media and AGS and HEK-Blue transfected cells were stimulated for 1 h with *H.* *pylori* at the MOI indicated in the absence of antibiotics, the media was replaced with plain antibiotic-free media, and cells were incubated for an additional 16 h. For LPS stimulation assays, AGS transfected cells were stimulated with 100 ng/ml LPS (InvivoGen) for 16 hrs. Cell culture supernatants were collected and stored at −80 °C until required for IL-8 quantification by ELISA using the BD OptEIA human IL-8 ELISA kit (BD Biosciences) and measuring absorbance using a plate reader (CLARIOstar, BMG Labtech) as previously.^16^^,^^23^ HEK-Blue cell culture supernatants were assayed for secreted alkaline phosphatase (SEAP) activity using QUANTI Blue solution (InvivoGen, USA), and SEAP activity was measured using a plate reader (CLARIOstar, BMG Labtech) as previously.^19^

NF-ĸB luciferase reporter assays were performed as previously.^16^^,^^24^ In brief, 1 × 10^5^ HEK293 cells were seeded in 24 well cell culture plates. The following day, each well was transfected with a total of 1150 ng of DNA, consisting of 60 ng/well Igκ luciferase plasmid,^16^ 50 ng/well dTK *Renilla* plasmid (Promega) and either 300 ng/well of a pIRES vector expressing IL-37b^12^ or 300 ng/well of control pIRES vector lacking the IL-37b insert (transfection control).^12^ The total DNA transfected per well was adjusted to 1150 ng using pcDNA_3._^16^ Cells were transfected using Lipofectamine 2000 reagent according to manufacturer’s instructions (Invitrogen) in a final volume of 1 ml of culture medium for 24 h. The following day, culture media was replaced and transfected HEK293 cells were stimulated for 4 h with *H.* *pylori* at the MOI indicated, and luminescence was measured using a plate reader (BMG Labtech).

### Chemical inhibition co-culture assays

For caspase and NOD1 inhibition experiments, AGS cells were pre-treated with either a pan-caspase inhibitor (Z-VAD-FMK, 20 μM, R&D systems), caspase-1 inhibitor (Z-WEHD-FMK, 20 μM, R&D systems), NLRP3 inhibitor (MCC950, 50 μM, Sigma Aldrich), or NOD1-specific inhibitor (ML130, 5 μM, Tocris) for 1 h before stimulation with *H.* *pylori* in antibiotic free media. Following 1 h of *H.* *pylori* stimulation, media was removed and cells were incubated for a total of 24 h in plain cell culture media supplemented with inhibitors. AGS cell viability in response to treatment with chemical inhibitors was determined by MTT assay (3-(4,5-dimethylthiazol-2-yl)-2,5-diphenyltetrazolium bromide, Abcam).

### SDS-PAGE and Western blot analysis of IL-37

AGS cell culture supernatants were collected and treated with protease inhibitors (cOmplete protease inhibitor cocktail, Roche), centrifuged (4,000g, 5 min, 4 °C) to remove cell debris and supernatants were subsequently concentrated using Amicon filters with a 10 kDa cut-off as per manufacturer’s instructions (Merck Millipore). For the detection of intracellular IL-37, AGS cells were lysed using lysis buffer (NaCl (4 M), Tris (1 M), glycerol (10% v/v), Triton X 100 (5% v/v), EDTA (100 mM), Na_3_VO_4_ (100 mM) and beta-glycerophosphate). Proteins contained in AGS cell supernatants and lysates were separated by SDS-PAGE using precast 4-12% Bis-Tris SDS gels (NuPAGE, Invitrogen) and transferred onto a PVDF membrane (Hybond ECL, Amersham Biosciences). The PVDF membranes were blocked with blocking buffer consisting of 1% BSA in TBST (TBS-Tween (0.05% (v/v)) for 1 h at room temperature. For the detection of IL-37, membranes were probed with monoclonal mouse anti-human IL-37 antibody (1:500 dilution, clone 37D12, eBioscience) overnight at 4 °C. Membranes were washed in TBST and incubated with secondary antibody (goat anti-mouse IgG (H+L)-HRP conjugate, polyclonal, Invitrogen) diluted 1:1,000 in blocking buffer for 1 h at room temperature. For the detection of actin, membranes were probed with mouse anti-beta actin monoclonal antibody (1:5,000, clone 15G5A11/E2, Thermo Fisher), washed and incubated with goat anti-mouse IgG-HRP conjugate (1:1,000, Invitrogen). The membranes were washed in TBST and developed using Clarity™ Western ECL Blotting Substrate (BioRad). Protein bands were quantified by densitometry using ImageStudioLite software (LI-COR Biosciences). For the analysis of intracellular IL-37 levels, data were normalized to total actin, taken as a loading control, and represented as fold.

### Proteomics: in-gel separation and sample preparation

Cell supernatant from AGS cells stimulated with *H.* *pylori* was collected, separated by SDS-PAGE, and gels were stained using Coomassie Brilliant Blue. Bands of approximately 17 kDa and 25 kDa were individually excised from SDS-PAGE gels and analyzed separately (*n* = 3). Individual gel sections were destained (50% (v/v) acetonitrile, ACN), and reduced with 2 mM tri(2-carboxyethyl)phosphine hydrochloride (Sigma-Aldrich, C4706) at 22 °C for 4 h on gentle rotation, alkylated by treatment with 25 mM iodoacetamide (Sigma-Aldrich) for 30 min, and digested with trypsin (Promega, V5111) 1:50 enzyme-to-substrate ratio at 37 °C for 18 h. Subsequently, peptides were extracted as previously,^25^ using serial 30-50% ACN in 0.5% (v/v) formic acid (FA), lyophilized and purified using reverse-phase C18 StageTips (Sep-Park cartridges, Waters, MA) in 85% (v/v) ACN in 0.5% (v/v) formic acid (FA), and centrifuged at 16,000g for 15 min. Peptides were lyophilized and acidified in 0.1% FA, 2% ACN, and quantified (Fluorometric Peptide Assay (23290, Thermo Fisher Scientific).

### Proteomic liquid chromatography-tandem mass spectrometry

Peptides were analyzed using previously established methods,^25^ using a nanoflow UPLC instrument (Ultimate 3000 RSLCnano, Thermo Fisher Scientific) that was coupled on-line to a Q-Exactive HF Orbitrap mass spectrometer in positive acquisition mode (Thermo Fisher Scientific) with a nanoelectrospray ion source (Thermo Fisher Scientific). For label-free proteomic analysis, peptides were loaded (Acclaim PepMap100, 5 mm × 300 µm i.d., µ-Precolumn packed with 5 µm C18 beads, Thermo Fisher Scientific) and separated (BioSphere C18 1.9 µm 120 Å, 360/75 µm × 400 mm, NanoSeparations) with a linear gradient from 2-100% (v/v) phase B (0.1% (v/v) FA in 80% (v/v) ACN) over 90 min or 120 min (2–100% 0.1% FA in acetonitrile) at a flow rate of 250 nl/min at 55°C. An MS1 scan was acquired from 300–1,500 *m*/*z* (60,000 resolution, 3 × 10^6^ AGC, 30-ms injection time) followed by MS/MS data-dependent acquisition with higher-energy collision-induced dissociation (HCD) and detection in the orbitrap (60,000 resolution, 1 × 10^5^ AGC, 110-ms injection time, 30% normalized collision energy, 1.4 *m/*z quadrupole isolation width). Multi-notch isolation of the top 7 most intense MS/MS ions from 300–2,000 *m*/*z* excluding the precursor ion and neutral loss clusters. Dynamic exclusion was activated for 25 sec. Data was acquired using Xcalibur software v4.0 (Thermo Fisher Scientific). All proteomic data have been deposited in PeptideAtlas Consortium via the PeptideAtlas proteomics repository: PASS01395.

### Proteomic data analysis

Proteomic data analysis was performed as previously.^26^ Peptide identification and quantification were performed using MaxQuant (v1.6.0.16) with its built-in search engine Andromeda.^27^ Tandem mass spectra were searched against a combined human reference proteome (71,798 entries, downloaded 10-2018) and *H.* *pylori* reference proteome (1,553 entries, UniProt, UP000000429, downloaded 1-2018) supplemented with common contaminants. Search parameters included carbamidomethylated cysteine as fixed modification and oxidation of methionine and *N*-terminal protein acetylation as variable modifications. Data was processed using either trypsin/P as the proteolytic enzyme with up to 2 missed cleavage sites allowed, or unspecific search. Precursor tolerance was set to ±4.5 ppm, and fragment ion tolerance to ±10 ppm. Results were adjusted to 1% false discovery rate (FDR) on peptide spectrum match (PSM) level (5% FDR for unspecific approach) employing a target-decoy approach at the peptide and protein levels. The label free quantification (LFQ) algorithm in MaxQuant was used to obtain quantification intensity values.

### *H.* *pylori* infection of mice

All mouse experiments were approved by the Monash Medical Center Animal Ethics Committee A (MMCA 2016/31). The generation of IL-37tg and IL-37tgxIL-1R8KO mice has been described earlier.^12^^,^^14^ Wild type C57BL/6, IL-37tg and IL-37tgxIL-1R8KO 8-12 week old female mice, all bred at Monash Medical Center, were anesthetised with isoflurane and infected with approximately 1 × 10^8^ *H.* *pylori* SS1 in 200 μl of PBS by oral gavage, or given 200 μl of PBS as a control, and were analyzed 3 months post infection, using our established techniques.^16^^,^^28^^,^^29^ To determine *H.* *pylori* colonization levels and gastritis of infected mice, mouse stomachs were opened along the greater curvature, washed in PBS and cut in half to include the greater curvature. Half of the stomach was used to quantify *H.* *pylori* colonization by enumerating the number of colony forming units (CFU) per gram of stomach tissue,^28^ and the other half was used for hematoxylin and eosin staining and to grade the level of chronic and acute gastritis^28^ using the Sydney System of grading gastritis.^30^ Chronic gastritis was used to define mononuclear cell infiltration into the gastric tissue, and acute gastritis referred to neutrophil cell infiltration in gastric tissues. The following six-point scale was used to define mononuclear cell infiltration: 1, mild multifocal; 2, mild widespread or moderate multifocal; 3, mild widespread and moderate or severe multifocal; 4, moderate widespread; 5, moderate widespread and severe multifocal; 6, severe widespread. Mouse serum was collected and stored at -80 °C until analyzed for antibody responses to *Η. pylori* infection.

### *H.* *pylori* ELISAs

Mouse serum *H.* *pylori* specific IgG antibody levels were determined by ELISA using a modification of a previously described method.^29^ Briefly, MaxiSorp immunoplates (NUNC) were coated with 250 μg/ml *H.* *pylori* SS1 sonicate and were incubated with serially diluted mouse sera. Bound antibodies were detected using a goat anti-mouse IgG-HRP conjugated secondary polyclonal antibody (1:2,000, Thermo Fisher) and the plates were developed using TMB substrate solution (3,3′,5,5′-Tetramethylbenzidine, Life Technologies, USA). Antibody titers were expressed as the reciprocal dilution of serum that gave an optical density at 450 nm five times the value of background. Mouse sera with known specific *H.* *pylori* antibody titers and from non-infected animals were included as a positive and negative controls, respectively on all ELISA plates.

### Isolation and activation of human primary lymphocytes

All experiments using human immune cells were approved by the Florence Ethics Committee code: BIO 114.013_AOUC. Primary lymphocytes were obtained from buffy coats of healthy adults. Informed consent was obtained from all donors according to the Helsinki Declaration. T and B cells from peripheral blood of healthy donors were purified by negative selection using the RosetteSep human T cell or human B cell enrichment cocktail (StemCell Technologies), followed by density gradient centrifugation on Lympholyte® Cell Separation Media (Cedarlane Laboratories). The purity of T cell and B cell populations was >90%, as assessed by flow cytometry.

### Stimulation and activation of PBMCs and primary human T and B cells

PBMCs were either left untreated or treated with 10 ng/ml of monomeric recombinant IL-37 (rIL-37),^31^ and stimulated with 1 mg/ml of LPS (InvivoGen) for 48 h as described previously.^31^ IL-6 was then measured by ELISA (R&D, Minneapolis, USA).

Primary human T or B cells were left untreated or treated with 10 ng/ml of monomeric recombinant IL-37 for 24 h and were activated by TCR/CD3 or IgM cross-linking by incubating the T and B cells with OKT3 (IgG from OKT3 (anti-CD3ε) hybridoma supernatants were purified using Mabtrap (GE Healthcare, Milan, Italy) and titrated by flow cytometry) or the goat anti–human immunoglobulin M (IgM) antibodies (Jackson Immunoresearch Laboratories), respectively, for 5 min at 37 °C. T and B cells were stained with FITC anti-human CD69 (Biolegend) or FITC anti-human CD86 (BD Pharmingen), respectively and analyzed by flow cytometry using a Guava easyCyte cytometer (Millipore). Data were analyzed and plotted using FlowJo Version 6.1.1 software (TreeStar).

For CFSE proliferation studies, proliferation was measured by flow cytometry using the CFSE dilution method.^9^ Briefly, B and T cells were stained with 10 µM CFSE (Molecular Probes) for 8 min at RT before stimulation with OKT3 (anti-CD3; American Type Culture Collection) cross‐linking on secondary antibody coated plates as described^9^ for T cells, or with anti-IgM (Jackson Immunoresearch) for B cells, and cells treated or not with rIL-37 (10 ng/ml). Cells were analyzed 72 h after activation by flow cytometry.

### Detection of Erk and Vav phosphorylation by Western blot

For the analysis of phosphorylated Erk1/2 and Vav, cells (5 × 10^6^/sample) were lysed in 20 mM Tris-HCl (pH 8, 150 mM NaCl, 1% Triton X-100 plus protease inhibitors). Rabbit polyclonal antibodies recognizing the phosphorylated forms of Vav1 (Y160) and Erk1/2 (T202/Y204) were purchased from Invitrogen and Cell Signaling, respectively. Mouse monoclonal antibodies against actin were purchased from Millipore and secondary peroxidase-labeled antibodies from Amersham Pharmacia Biotech. Erk1/2 and Vav phosphorylation was determined by chemiluminescence (SuperSignal; West Pico Chemiluminescent Substrate kit; Pierce Chemical), scanned using a laser densitometer (Duoscan T2500, Agfa) and quantified with ImageQuant 5.0 software (Molecular Dynamics). Data were normalized referring to total Erk1/2 as a loading control.

### Migration and polarization assays

Chemotaxis assays were performed using 24 well Transwell chambers with 5 µm pore polycarbonate membranes (Corning Life Sciences, Schiphol-Rijk, The Netherlands) as previously described.^32^^,^^33^ Briefly, filters were soaked overnight in RPMI containing 1% BSA. The chemotaxis medium (500 µL of RPMI 1% BSA) with or without 100 nM CXCL12 (Sigma-Aldrich, St. Louis, MO, USA) was placed in the lower chamber, and 100 µL of the cell suspension (5 × 10^5^ cells/sample) either untreated or pre-treated with rIL-37^10^ (10 ng/ml) was placed in the upper chamber. After 3 h of incubation at 37 °C in humidified air with 5% CO_2_, the upper chamber was emptied, filters were removed, and the cells in the lower chamber were counted by flow cytometry. The migration index was calculated by determining the ratio of migrated cells in treated versus untreated samples. Human primary T cells were stained with anti-CD3 antibodies (eBioscience, San Diego, CA, USA) before analysis.

For cell polarization assays, diagnostic microscope slides were coated with 10 μg/ml fibronectin and T cells (1 × 10^5^/sample), pre-treated or not with recombinant IL-37 (IL-37, 10 ng/ml), were allowed to adhere for 5 min before stimulation for 5 min with 100 ng/ml CXCL12. Slides were immediately fixed in 4% paraformaldehyde at RT for 20 min, washed for 5 min in PBS and incubated with TRITC-labeled Phalloidin for 1 h at RT. Confocal microscopy was performed using a Zeiss LSM700 (Carl Zeiss, Jena, Germany). Images were acquired with pinholes opened to obtain 0.8 mm-thick sections. Detectors were set to detect an optimal signal below the saturation limits. Images were processed with Zen 2009 image software (Carl Zeiss).

### Analysis of immune synapses

Analysis of immune synapses was performed as previously.^34^ For immune synapse (IS) analyses, human primary B cells or human primary T cells or both were treated with rIL-37 10 ng/ml for 24 h. Human primary B cells (used as antigen presenting cells (APCs)) were washed and pulsed for 2 h with 10 µg/ml SEE/SEB. APCs were then washed, mixed with primary T cells in a 1:1 ratio for 15 min at 37 °C and then plated on poly-L-lysine coated wells of diagnostic microscope slides (Erie Scientific Company). Cells were allowed to adhere for 15 min and then fixed in 4% paraformaldehyde at RT for 20 min. Antigen independent conjugates were obtained by mixing T cells and APCs in the absence of SEE/SEB. Following fixation, samples were washed 5 min in PBS and incubated with an anti-P-Tyr antibody (clone 4G10, Millipore) for 1 h at RT. After washing in PBS, samples were incubated for 1 h at room temperature with Alexa Fluor 488 labeled goat anti-mouse IgG (Life technologies) secondary antibody. Confocal microscopy was performed using a Zeiss LSM700 (Carl Zeiss, Jena, Germany). Images were acquired with pinholes opened to obtain 0.8mm-thick sections. Detectors were set to detect an optimal signal below the saturation limits. Images were processed with Zen 2009 image software (Carl Zeiss) and analyses were performed using ImageJ software. Polarization of P-Tyr at the immune synapse site was determined based on the presence of fluorescent P-Tyr staining directly at the T-APC contact site. The percentage of P-Tyr present within a sample was determined by expressing the number of conjugates with synaptic staining versus the total number of conjugates present within a sample as a percentage.

### Inhibition of *H.* *pylori*-specific T cell proliferation by IL-37

After approval by the local ethics committee, following informed consent, T cells specific for *H.* *pylori* were derived from the stomach of ten *H.* *pylori*-infected patients (5 males, 5 females, mean age 67), as previously described.^35^ Briefly, biopsy specimens were cultured for 7 days in RPMI 1640 medium supplemented with human IL-2 (50 U/mL; Eurocetus, Milan, Italy) to expand *in vivo*–activated T cells. Each T cell line was screened for responsiveness to *H.* *pylori* by measuring [^3^H] thymidine uptake after 60 h of stimulation with medium, or *H.* *pylori* lysate (aqueous extract of NCTC11637 strain, 10 μg/mL) in the presence of irradiated autologous APCs. We then analyzed in a dose–dependent fashion the effects of rIL-37 (0.1−10 ng/ml) on *H.* *pylori*-induced proliferation of T cells (10 × 10^4^) by measuring [^3^H]thymidine uptake after 60 h of co-culture with irradiated autologous APCs (5 × 10^4^) in the presence of medium, or *H.* *pylori* lysate (10 μg/ml).

### Statistical analyses

Data were analyzed using GraphPad Prism software V9. All data were analyzed using either t-test, Mann-Whitney Test, one way Ordinary One-way ANOVA with Tukey’s or Dunnett’s multiple comparison’s test, or Two-way ANOVA as indicated.

## Results

### IL-37 is produced and secreted by gastric epithelial cells in response to *H.* *pylori* infection

**Figure 1.**
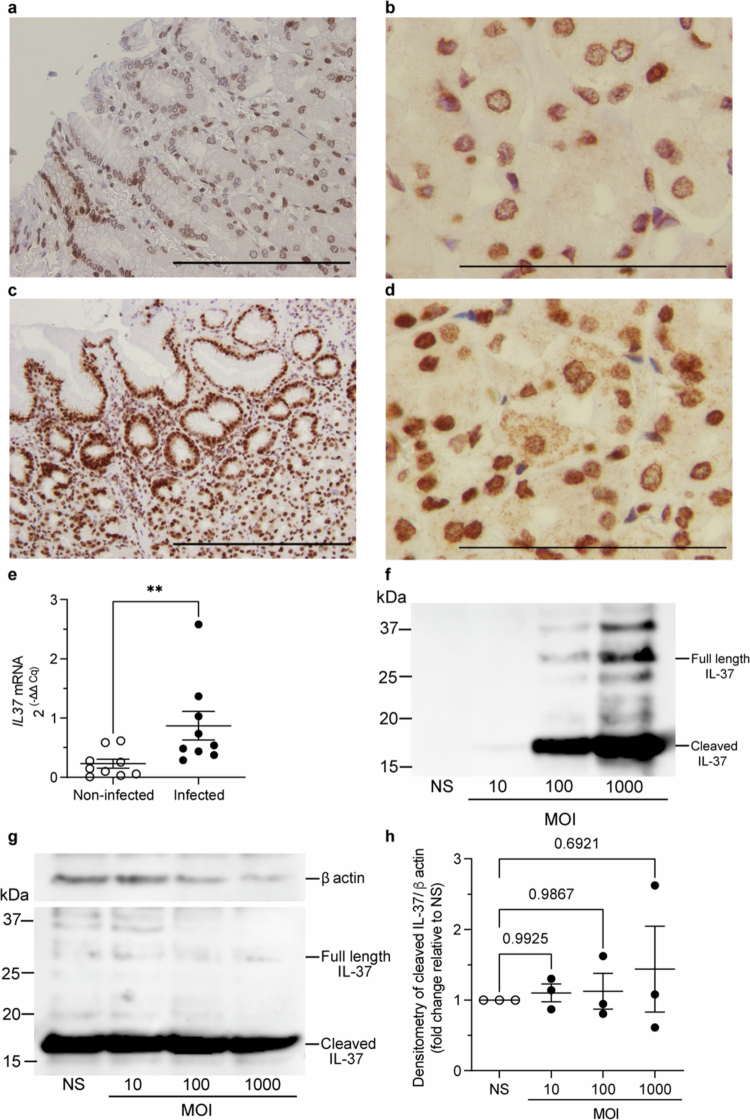
*H.* *pylori* induces IL-37 production and secretion in human gastric biopsies and human gastric epithelial cells. (a) Immunohistochemistry of IL-37 in human gastric biopsies obtained from (a, b) non-infected or (c, d) *H.* *pylori*-infected individuals. Scale bars represent 20 µm (a, c) or 10 µm (b, d). Representative images from *n* = 9 individuals per group. (e) qRT-PCR of *IL37* expression in human gastric biopsies obtained from non-infected (open circles) and *H.* *pylori-*infected (filled circles) individuals. Data are mean ± standard error of the mean (SEM) of *n* = 9 individuals per group. Statistical significance determined by Mann-Whitney Test. **P = 0.0056. IL-37 (f) secreted into cell culture supernatants or (g) detected in cell lysates obtained from AGS cells stimulated with *H.* *pylori* 251 for a total of 24 h at an increasing MOI, or non-stimulated (NS) as controls was detected by immunoblotting. Detection of β-actin in the cell lysates (g) was used as a loading control. Cleaved and full-length IL-37 are indicated. Representative of *n* = 3 experiments. (h) Densitometry of blots shown in G, showing cleaved IL-37 protein, relative to β-actin present in the cell lysates of AGS cells stimulated with an increasing MOI of *H.* *pylori* 251 (closed circles), and represented as fold change relative to non-stimulated (NS, open circles) cells. Shown are individual data points of *n* = 3 biological replicates with mean ± SEM. Statistical significance determined by One-way ANOVA with Dunnett’s multiple comparison test. *P* values are shown.

We hypothesized that *H.* *pylori* can induce the production of IL-37 and exploit the pan-immunosuppressive properties of IL-37 to prevent the development of an effective host immune response. To test this hypothesis, we examined the presence and abundance of IL-37 in gastric biopsies obtained from *H.* *pylori-*infected and non-infected individuals using immunohistochemistry. We found that IL-37 protein was detected at low abundance and was predominantly nuclear in biopsies obtained from non-infected individuals (Figure 1a,b). In contrast, IL-37 was highly abundant in *H.* *pylori* positive gastric biopsies, localizing to both the nucleus and cytoplasm in epithelial cells and only to the nucleus in lymphocytes (Figure 1c,d). Furthermore, we quantified *IL37* mRNA expression levels in all gastric biopsies obtained from non-infected and *H.* *pylori* infected individuals by qRT-PCR. We found that *IL37* was significantly increased in the gastric tissue of *H.* *pylori*-infected individuals compared to non-infected controls (Figure 1e, *P* = 0.0056), indicating that *H.* *pylori* increased gastric IL-37 in infected individuals.

As *H.* *pylori* infection was associated with a striking increase in IL-37 expression within gastric tissues, we next aimed to confirm the ability of *H.* *pylori* to increase IL-37 production and secretion by human gastric epithelial cells. As IL-37 is a “dual-function” cytokine with intracellular and extracellular activities,^12^^,^^15^ we examined IL-37 protein within and secreted by human gastric adenocarcinoma epithelial cells (AGS cells) in response to *H.* *pylori* infection. There are five isoforms of IL-37 (termed IL-37a-e) ranging from 17-25 kDa in size, with the 17 kDa caspase-cleaved and mature form of IL-37b being the most active.^36^^-^^39^ We found that *H.* *pylori* induced the secretion of predominantly the 17 kDa mature form of IL-37 by AGS cells in a dose-dependent manner, designated as ‘cleaved IL-37’ (Figure 1f) to significant levels compared to non-infected AGS controls (Supplementary Figure S1). In addition, we observed no significant changes to intracellular IL-37 abundance in response to *H.* *pylori* infection (Figure 1g,h), and with minimal IL-37 secretion in the absence of infection (Figure 1f). We confirmed AGS cells remained viable when infected with *H.* *pylori* 251 bacteria at an MOI 1, 10 and 100, with AGS cells viability decreasing when infected at an MOI of 1,000 (Supplementary Figure S2). These findings support that AGS cells infected with *H.* *pylori* at either an MOI of 1, 10 or 100 remained viable and that IL-37 secretion into cell culture supernatants at these infectious doses was not attributed to host cell death. We confirmed the identity of the 17 kDa IL-37b protein secreted by *H.* *pylori-*stimulated AGS cells following gel excision, using mass spectrometry-based proteomics **(Supplementary Table S1)**, in addition to a number of higher molecular weight isoforms of IL-37, consistent with IL-37 homodimers,^39^ and including a protein of approximately 25 kDa corresponding to ‘full-length’ IL-37 (Figure 1f). Collectively, these findings reveal that IL-37 is expressed at low levels during steady-state by human gastric epithelial cells from non-infected individuals, and its production and secretion is markedly increased in response to *H.* *pylori* infection.

### Gastric epithelial cells secrete IL-37 in response to *H.* *pylori* infection in a TLR, NOD1, cagPAI and caspase-dependent manner

**Figure 2.**
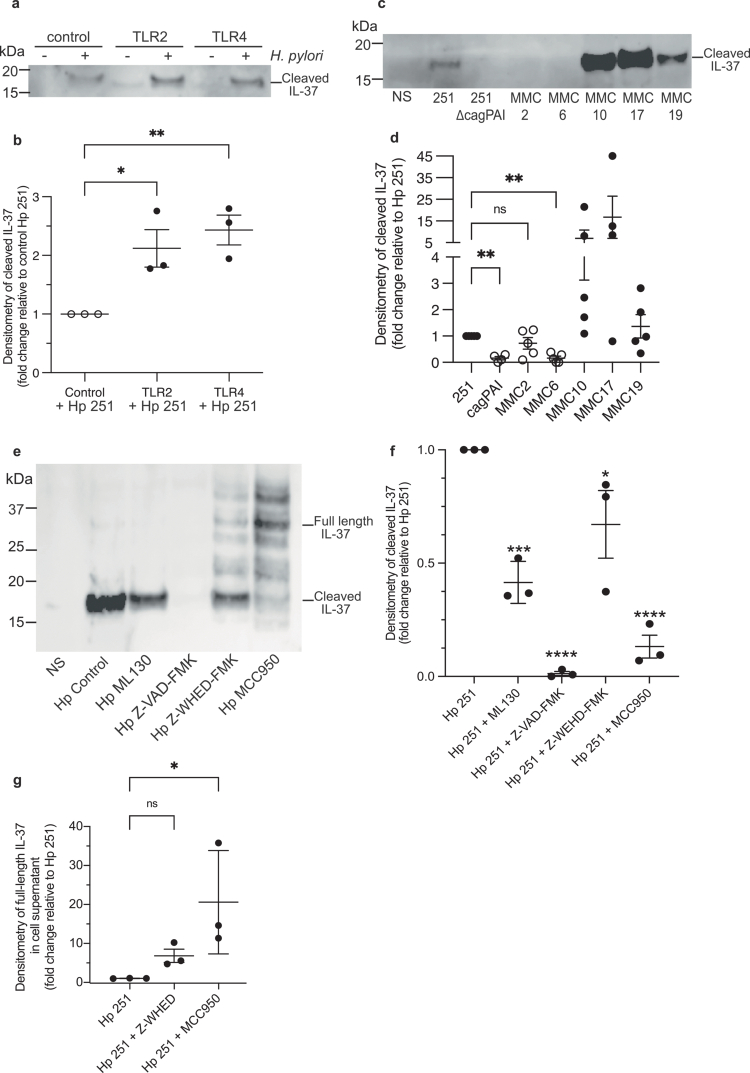
Gastric epithelial cells secrete IL-37 in response to *H.* *pylori* stimulation in a TLR, cagPAI, NOD1 and caspase-dependent manner. (a) AGS cells transiently transfected with either a TLR2, TLR4 or control plasmid (control), were stimulated with *H.* *pylori* 251 (+, MOI 100) or not stimulated as a control (−). IL-37 in supernatants was detected by immunoblotting 24 h post stimulation. Representative of *n* = 3 biological replicates. (b) Densitometry of IL-37 secreted by the three biological replicates of TLR2, TLR4 and control transfected AGS cells in response to *H.* *pylori* stimulation; fold-change in secreted IL-37 relative to *H.* *pylori* stimulated transfection control cells is depicted. Symbols are individual data points, lines indicate means ± SEM. (c) Detection of IL-37 secreted by AGS cells stimulated with either *H.* *pylori* 251 (251), *H.* *pylori* ΔcagPAI (cagPAI), or *H.* *pylori* isolates MMC2, MMC6, MMC10, MMC17 or MMC19 at an MOI of 100 for a total of 24 h. Data are representative of n ≥ 4 biological replicates. (d) Densitometry of n ≥ 4 biological replicates represented in panel c. Symbols show individual data points, lines show mean ± SEM. (e) AGS cells pre-treated for 1 h with inhibitors of NOD1 (ML130, 5 mM), NLRP3 (MCC950, 50 μM), caspase-1 (Z-WEHD-FMK, 20 μM) or pan-caspase (Z-VAD-FMK, 20 μM) or DMSO (Hp control) as vehicle were stimulated with *H.* *pylori* 251 (MOI 100) for 24 h in the presence of inhibitors. IL-37 secreted into the cell supernatant was then detected by immunoblotting. Data are representative of *n* = 3 biological replicates. Densitometry of (f) full length or (g) cleaved IL-37 in the cell supernatants of *n* = 3 biological replicates represented in panel e. Fold decrease is relative to stimulation with *H.* *pylori* 251 DMSO controls (Hp 251). Data are the mean ± standard error of the mean (SEM) of *n* = 3 biological replicates. Statistical significance determined by One-way ANOVA with Dunnett’s multiple comparison test. *P < 0.05, **P < 0.01, ***P < 0.001, ****P < 0.0001. ns = not significant.

As IL-37 is produced by host cells in response to TLR activation^12^ and is capable of supressing TLR responses and particularly TLR4 responses to LPS,^12^ we next investigated the mechanisms of TLR-mediated IL-37 secretion in response to *H.* *pylori* infection. *H.* *pylori* can be detected by TLR2 and TLR4,^40^ with *H.* *pylori* LPS modifications rendering detection of its LPS atypically via TLR2 and not TLR4^7^. To examine the mechanisms of TLR-mediated IL-37 production, we overexpressed TLR2 and TLR4 individually in AGS cells which lack functional TLR2,^41^ or TLR4 activity due to lacking expression of the MD-2 co-receptor^41^ and examined their contribution to *H.* *pylori*-mediated IL-37 secretion. *H.* *pylori* stimulation of TLR2- or TLR4-overexpressing AGS cells increased IL-37 secretion 2-fold compared to transfection controls (Figure 2a,b, *P* < 0.05, *P* < 0.01 respectively), demonstrating that these TLRs contribute to IL-37 release by gastric epithelial cells in response to *H.* *pylori* infection.

Another key host PRR responsible for the detection of *H.* *pylori* is nucleotide-binding oligomerization domain-containing protein 1 (NOD1), an intracellular PRR that detects Gram-negative bacterial peptidoglycan introduced into host epithelial cells via a type 4 secretion system (T4SS) encoded by the *H.* *pylori* cagPAI.^17^ We identified the contribution of both NOD1 and the *H.* *pylori* cagPAI to IL-37 secretion by gastric epithelial cells, as IL-37 secretion was increased in AGS cells stimulated with a *H.* *pylori* cagPAI positive strain (251) compared to the cagPAI isogenic mutant (251 ∆cagPAI) (Figure 2c,d, *P* < 0.01). We next screened a range of *H.* *pylori* clinical isolates for the presence of the cagPAI by sequencing (**Supplementary Figure S3)** and used these strains to further determine the contribution of cagPAI in mediating IL-37 secretion by AGS cells (Figure 2c,d). *H.* *pylori* cagPAI positive isolates (MMC 10, 17 and 19) induced up to 15-fold more IL-37 secretion by AGS cells than cagPAI negative strains (MMC 2 and 6) as detected by Western blot, further supporting the contribution of the cagPAI in facilitating IL-37 secretion by gastric epithelial cells (Figure 2c,d). Furthermore, the *H.* *pylori* MMC2 isolate that has a partial cagPAI deletion **(Supplementary Figure S3)**, induced IL-37 secretion to a comparable level as that produced by the wild-type *H.* *pylori* 251 strain (Figure 2c,d). Genetic examination of *H.* *pylori* MMC2 revealed that it contained the cagA gene, suggesting that cagA, in addition to cagPAI and NOD1, may also contribute to IL-37 secretion by host cells **(Supplementary Figure S3)**. Furthermore, the ability of these clinical isolates to induce IL-37 secretion was examined using human gastric mucosoids, a model of human gastric organoids grown in a monolayer with an apical mucus layer at the air-liquid interface. Gastric mucosoids were generated from three healthy human subjects and stimulated with either *H.* *pylori* clinical isolates, *H.* *pylori* 251 or *H.* *pylori* 251 cagPAI, and IL-37 secreted into the supernatant was quantified **(Supplementary Figure S4)**, We show that IL-37 was significantly secreted by gastric mucosoids in response to infection with all of the *H.* *pylori* clinical isolates examined, in addition to in response to infection with *H.* *pylori* 251, compared to non-infected mucosoid controls (*P* < 0.0001). Similarly, no significant amount of IL-37 was detected in the supernatants obtained from mucosoids infected with *H.* *pylori* 251 Δ*cag*PAI isogenic mutant, compared to non-infected controls, further confirming the contribution of the cagPAI to the production of IL-37 by gastric epithelial cells **(Supplementary Figure S4)**. In addition, infection of mucosoids with a *H.* *pylori* 251 ∆CagA isogenic mutant suggested a trend in the ability of this strain to induce IL-37 production, compared to the *H.* *pylori* 251 cagPAI mutant and non-stimulated controls **(Supplementary Figure S4)**, and therefore the contribution of cagA to IL-37 production was further investigated.

To determine the contribution of *H.* *pylori* CagA to IL-37 production, we next examined the ability of the *H.* *pylori* 251 ∆CagA isogenic mutant to induce the production of IL-37 in AGS cells by Western blot **(Supplementary Figure S5)**, We found that *H.* *pylori* 251 ∆CagA induced significant levels of IL-37 secretion by AGS cells compared to non-stimulated control cells and AGS cells stimulated with *H.* *pylori* 251 ∆cagPAI (*P* < 0.0001), as detected by Western blot and confirmed by densitometry **(Supplementary Figure S5)**. However, the ability of the cagA mutant to induce IL-37 secretion by AGS cells was impaired when compared to *H.* *pylori* 251 wild type bacteria (*P* < 0.0001). These finding suggest that cagA contributes to, but is not the sole factor responsible for, the secretion of IL-37 by gastric epithelial cells in response to *H.* *pylori* infection.

The contribution of NOD in mediating IL-37 secretion in response to *H.* *pylori* infection was further examined by blocking NOD1 signaling in AGS cells using the NOD1 inhibitor ML130, which did not affect cell viability **(Supplementary Figure S6)**. *H.* *pylori* 251 stimulation of AGS cells treated with ML130 resulted in a significant reduction in IL-37 secretion compared to *H.* *pylori* stimulated control AGS cells (Hp 251) (Figure 2e,f, *P* < 0.001). Together, these findings indicate that NOD1 and the *H.* *pylori* cagPAI, in addition to TLR2 and TLR4, promote IL-37 secretion by gastric epithelial cells during *H.* *pylori* infection. This highlights that *H.* *pylori* utilizes multiple PRRs to induce IL-37 production during infection.

The mechanisms whereby IL-37 is secreted by epithelial cells are not fully understood. Similar to other IL-1 family cytokines, IL-37 does not contain a classical signal peptide. However, there is a caspase-1 cleavage site at aspartic acid 20 (D20) of IL-37, and caspase-1 is known to contribute to the cleavage and secretion of IL-37.^15^ To determine whether caspases are required for *H.* *pylori*-mediated secretion of cleaved IL-37, we treated AGS cells with either a caspase-1 inhibitor (Z-WHED-FMK), a selective inhibitor of the NLRP3 (NOD-like receptor protein-3) inflammasome that also prevents downstream activation of caspase-1 (MCC950), or a pan-caspase inhibitor (Z-VAD-FMK). None of the three inhibitors affected cell viability **(Supplementary Figure S6)**. We observed a significant reduction in cleaved IL-37 secreted by *H.* *pylori* stimulated AGS cells treated with either caspase-1 or NLRP3 inhibitors, compared to non-inhibitor controls (Figure 2e,f, *P* < 0.05, *P* < 0.0001 respectively). Furthermore, minimal IL-37 was secreted by *H.* *pylori* stimulated AGS cells upon treatment with the pan-caspase inhibitor Z-VAD-FMK (Figure 2e,f, *P* < 0.0001). In addition, the reduction in cleaved IL-37 secreted by *H.* *pylori* stimulated AGS cells treated with the NLRP3 inhibitor (MCC950) was associated with a greater than 10-fold increase in secreted full-length IL-37 (Figure 2e,g, *P* < 0.05). Full-length extracellular IL-37 has been reported to be able to suppress inflammation,^42^ suggesting that full-length IL-37 secreted in response to *H.* *pylori* stimulation in an NLRP3-independent manner may be functional. Taken together, these findings show that the NLRP3 inflammasome and caspases, including caspase-1, contribute to the secretion of cleaved IL-37 from *H.* *pylori* stimulated epithelial cells.

### IL-37 attenuates TLR2, TLR4 and NOD1-mediated activation and TLR-mediated IL-8 responses to H. pylori infection

**Figure 3.**
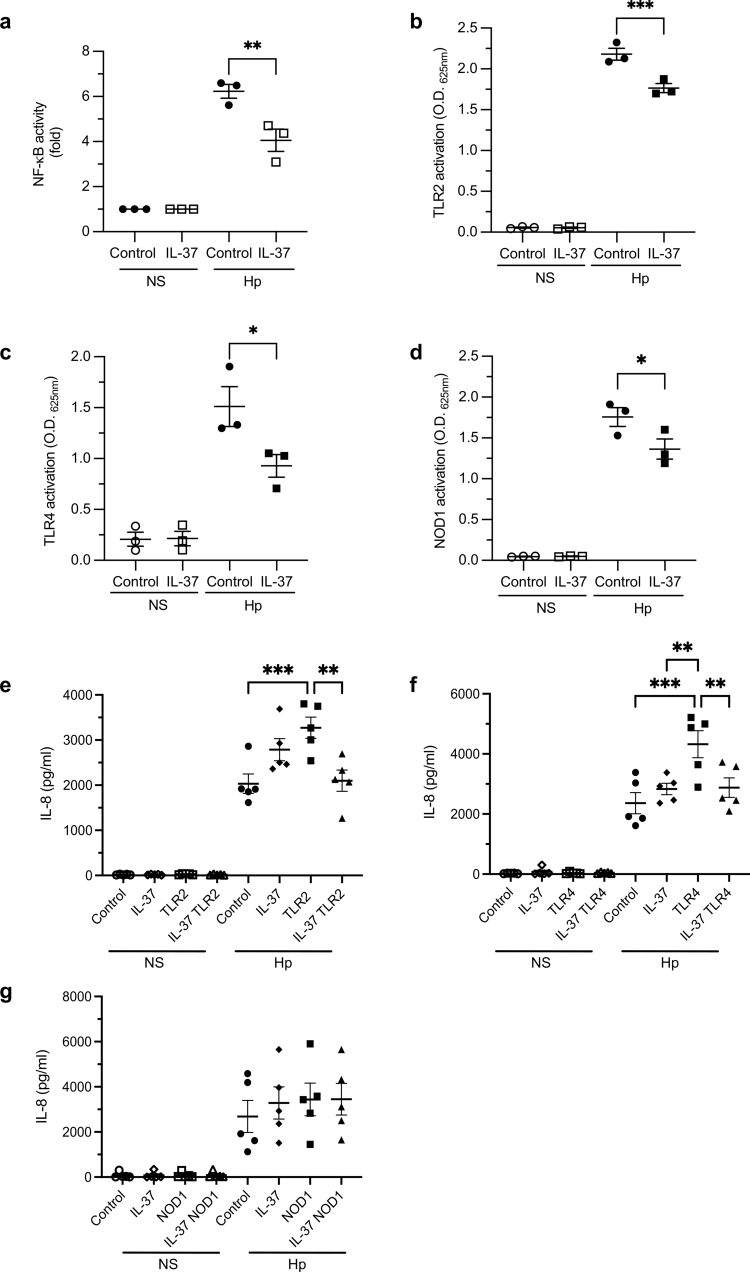
IL-37 reduces TLR and NOD1 mediated activation and TLR mediated IL-8 responses to *H.* *pylori* infection. (a) Fold NF-κB luciferase activity in HEK293 cells transfected with an IL-37-expression (IL-37) or control (control) vector and stimulated for 24 h with *H.* *pylori* 251 (Hp, MOI 100) or not stimulated (NS) as controls. Data are representative of *n* = 3 biological replicates. (b−d) HEK-Blue NF-κB reporter cells stably expressing either (b) TLR2, (c) TLR4 or (d) NOD1 were transiently transfected with an IL-37-expression (IL-37, squares) or control (control, circles) construct and stimulated with *H.* *pylori* 251 (Hp, MOI 100, filled shapes) or not stimulated (NS, open shapes) as controls. Data are the mean ± SEM of *n* = 3 biological replicates. (e−g) AGS cells were transiently transfected for 24 h with an IL-37 expression construct (IL-37), or a control construct (control, circles). Cells were also co-transfected with either a (e) TLR2, (f) TLR4 or (g) NOD1 expression construct as indicated. The following day, AGS cells were stimulated with *H.* *pylori* strain 251 (Hp, filled shapes) at an MOI of 100, or were not stimulated as controls (NS, open shapes), for 16 h. The level of IL-8 secreted into the cell supernatants was quantified by ELISA. Data are pooled of *n* = 5 independent experiments. Error bars represent mean ± SEM of biological replicates. All statistical significance was determined using One-Way ANOVA with Tukey’s multiple comparisons test. *P < 0.05, **P < 0.01, ***P < 0.001, ****P < 0.0001.

As IL-37 functions to reduce TLR-mediated cytokine responses in immune cells,^12^^,^^14^^,^^15^^,^^43^ we investigated whether IL-37 could reduce host PRR-mediated NF-ĸB and IL-8 responses during *H.* *pylori* infection. First, we examined whether IL-37 could suppress NF-ĸB activation in response to *H.* *pylori* infection. To do this, HEK293 cells were transfected with a pIRES-IL-37b expression plasmid or a control vector, in addition to an NF-ĸB luciferase construct, and NF-ĸB activity in response to *H.* *pylori* infection was examined (Figure 3a). We found that NF-ĸB activity was reduced in *H.* *pylori* stimulated IL-37b-expressing HEK293 cells, compared to vector control stimulated cells, suggesting that IL-37 suppressed NF-ĸB activation in response to *H.* *pylori* infection (Figure 3a, *P* < 0.01).

As we identified that IL-37 decreased *H.* *pylori* mediated NF-ĸB activation, we set out to determine which PRR-mediated responses were suppressed by IL-37 during *H.* *pylori* infection. To do this, HEK-Blue cells expressing a single PRR were transfected with either an IL-37b expression construct or a control vector, and activation of their PRR in response to *H.* *pylori* was determined. Transfection of IL-37b into HEK-Blue cells expressing either TLR2, TLR4 or NOD1 significantly reduced *H.* *pylori-*mediated PRR activation compared to vector control cells (Figure 3b-d, *P* < 0.001, *P* < 0.05, *P* < 0.05 respectively). Having confirmed the ability of IL-37 to suppress TLR4-mediated IL-8 production in TLR4-expressing AGS cells stimulated with LPS **(Supplementary Figure S7)**, we next sought to examine the ability of IL-37 to suppress TLR2, TLR4 and NOD1 mediated IL-8 production in AGS cells in response to *H.* *pylori* infection. We found that IL-37 significantly reduced TLR2 and TLR4-mediated IL-8 production by AGS cells in response to *H.* *pylori* infection (*P* < 0.001, *P* < 0.05 respectively), but not NOD1-mediated IL-8 responses (Figure 3e-g). Furthermore, expression of IL-37 alone did not suppress IL-8 production in response to *H.* *pylori* infection in AGS cells, revealing that IL-37 reduced IL-8 responses to *H.* *pylori* infection only when co-expressed with TLR2 or TLR4 (Figure 3e-g). Collectively, these findings identify that IL-37 functions to substantially suppress *H.* *pylori*-mediated TLR2, TLR4 and NOD1 activation, and TLR2- and TLR4-mediated IL-8 responses in epithelial cells, to ultimately limit the induction of innate immune responses in the host.

### IL-37 augments *H.* *pylori* colonization and impairs gastric inflammation and antibody responses in IL-37 transgenic mice

**Figure 4.**
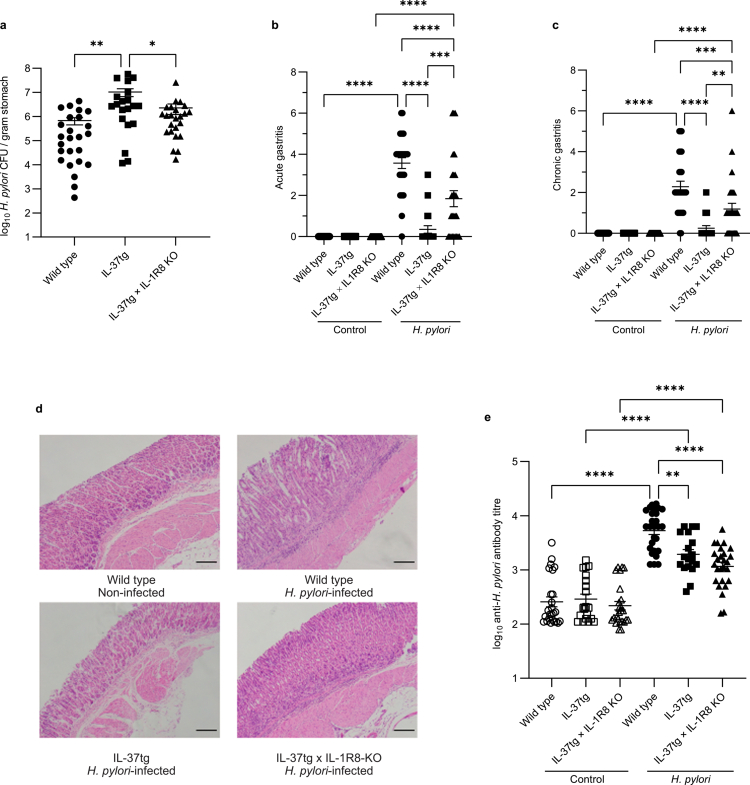
IL-37 augments *H.* *pylori* colonization and impairs chronic and acute gastric inflammation and antibody responses in mice. Groups of wild type C57BL/6 (circles), human IL-37b transgenic mice (IL-37tg, squares) and IL-37tg mice deficient in IL-1R8 (IL-37tg × IL-1R8KO, triangles) were infected with *H.* *pylori* SS1 (filled shapes), or not infected (open shapes) as controls for 3 months. (a) *H.* *pylori* colonization levels of infected wild type C57BL/6 (*n* = 25), IL-37tg (*n* = 20) and IL-37tg x IL-1R8 (*n* = 24) mice were determined 3 months post infection. Gastric biopsies from non-infected (Control) and *H.* *pylori* infected (*H.* *pylori*) wild type (*n* = 21, *n* = 25 respectively), IL-37tg (*n* = 17, *n* = 20) and IL-37tg × IL-1R8KO (*n* = 24, *n* = 25) mice were graded for (b) acute and (c) chronic gastritis. (d) Representative images of H&E stained gastric tissue obtained from non-infected and *H.* *pylori* infected wild type C57BL/6, and *H.* *pylori* infected IL-37tg and IL-37tg × IL-1R8KO mice. Scale bars represent 40 µm. (e) *H.* *pylori-*specific IgG antibody titers from non-infected (Control, open shapes) and *H.* *pylori* infected (*H.* *pylori*, filled shapes) C57BL/6 wild type (*n* = 25, *n* = 27 respectively), IL-37tg (*n* = 18, *n* = 20) and IL-37tg × IL-1R8KO (*n* = 24, *n* = 25) animals 3 months post-infection were determined by ELISA. All data are the mean ± SEM of individual animals pooled from *n* = 3 biological experiments. Statistical significance was determined using One-Way ANOVA with Tukey’s multiple comparisons test. *P < 0.05, **P < 0.01, ***P < 0.001, ****P < 0.0001.

Despite extensive efforts, a murine homolog of IL-37 remains unknown. Hence, mice transgenic for human IL-37b (IL-37tg mice) are routinely used for *in vivo* studies of IL-37 functions.^12^^,^^14^^,^^38^^,^^43^ Furthermore, we previously identified that the secreted form of IL-37 is detected by interleukin-1 receptor 8 (IL-1R8),^14^ which is expressed by several cell types including gastrointestinal epithelial cells.^44^ Hence, we generated IL-37tg mice deficient in IL-1R8 (termed IL-37tgxIL-1R8KO),^14^ to elucidate the contribution of IL-1R8 in mediating IL-37 functions. To investigate the effects of IL-37 on *H.* *pylori* mediated pathogenesis *in vivo*, we infected IL-37tg, IL-37tgxIL-1R8KO and C57BL/6 wild-type (WT) control mice which lack IL-37 with *H.* *pylori* SS1. The mouse colonizing strain *H.* *pylori* SS1 was able to induce significant IL-37 production by AGS cells compared to non-infected cells, and to comparable levels as the *H.* *pylori* 251 strain **(Supplementary Figure S8)**, and was therefore used to examine *H.* *pylori*-mediated IL-37 responses *in vivo*. Three months post-infection with *H.* *pylori* SS1, colonization levels were enumerated and found to be significantly elevated in IL-37tg mice compared to infected WT and IL-37tgxIL-1R8KO mice (*P* < 0.01, *P* < 0.05 respectively), whereas colonization was comparable between IL-37tgxIL-1R8KO and WT mice (Figure 4a). These findings indicate that IL-37 enhanced *H.* *pylori* colonization, and that the receptor IL-1R8 contributed to this effect.

It has been proposed that *H.* *pylori* modulates the host immune system, resulting in mild and chronic gastritis that is ineffective at clearing the infection.^2^ We hypothesized that IL-37 mediates chronic immunosuppression by impairing gastric immune cell recruitment and effective immune responses during *H.* *pylori* infection. To determine the effect of IL-37 on limiting gastric inflammation, we quantified the levels of acute and chronic gastritis in all non-infected and *H.* *pylori* infected animals 3 months post infection. Acute and chronic gastritis were defined by neutrophil and mononuclear cell infiltration into the gastric tissue, respectively. We found that IL-37 impaired immune cell recruitment to the gastric mucosa, as *H.* *pylori* infected IL-37tg mice had significantly reduced acute and chronic gastritis compared to infected IL-37tgxIL-1R8KO and WT mice, and similar levels of gastritis when compared to non-infected IL-37tg mice 3 months post-infection (Figure 4b-d**)**. Furthermore, both acute and chronic gastritis were reduced in *H.* *pylori-*infected IL-37tgxIL-1R8KO mice compared to infected WT mice (*P* < 0.0001, *P* < 0.001 respectively), suggesting that IL-37 may limit gastric immune cell migration and therefore gastritis via both IL-1R8-dependent and -independent mechanisms (Figure 4b-d).

Next, we examined the contribution of IL-37 to dampening *H.* *pylori* specific serum antibody responses. Despite all *H.* *pylori* infected mice being able to generate a *H.* *pylori*-specific antibody response compared to their non-infected controls, IL-37 expressing mice were impaired in their antibody responses (Figure 4e). Specifically, IL-37tg infected mice exhibited significantly impaired *H.* *pylori*-specific IgG responses compared to infected WT mice (*P* < 0.01), as did infected IL-37tgxIL-1R8KO mice, suggesting that IL-37 reduced *H.* *pylori*-specific antibody responses in an IL-1R8 independent manner (Figure 4e). Collectively, these findings show that IL-37 functions to enhance *H.* *pylori* colonization while limiting gastric inflammation and impairing *H.* *pylori*-specific immunity, and that IL-1R8 partially mediates these effects.

### IL-37 functions to limit human T cell and B cell activation to facilitate *H.* *pylori*-mediated immune suppression in the host

**Figure 5.**
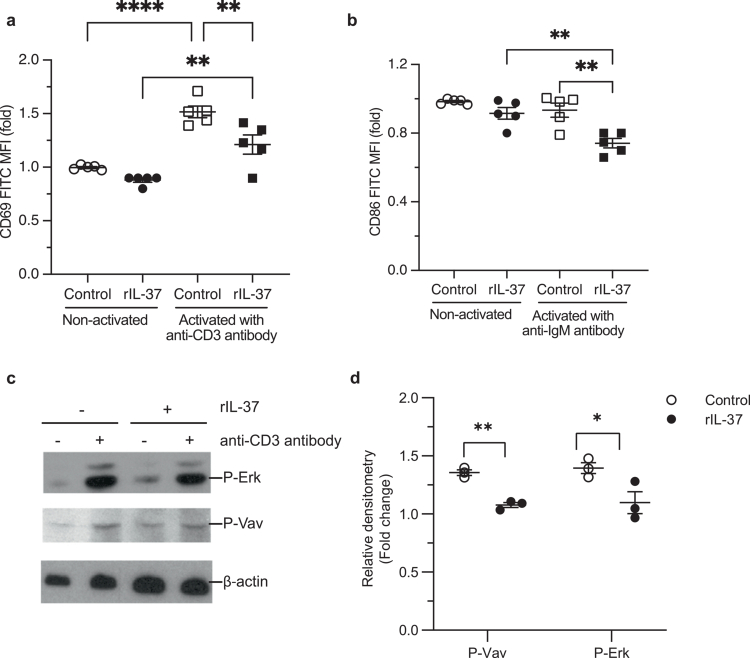
IL-37 functions to limit human T cell and B cell activation to facilitate *H.* *pylori* mediated immune suppression in the host. Expression of (a) CD69 and (b) CD86 on purified human peripheral blood T or B cells, respectively from five healthy donors treated with 10 ng/ml recombinant IL-37 (rIL-37, filled shapes) or not treated with IL-37 (control, open shapes) for 24 h and then stimulated with anti-CD3 (squares) or anti-IgM monoclonal antibodies (squares), respectively. Data are expressed as mean fluorescence intensity (MFI) ± SEM relative to non-treated cells, from individual donors. (c) Primary human T cells pre-treated with 10 ng/ml rIL-37 (rIL-37) or non-treated controls, were activated by T cell receptor cross-linking with anti-CD3 antibody and analyzed by immunoblot by staining for P-Erk, P-Vav, and actin. Shown is a representative image from *n* = 3 biological experiments. (d) Densitometry of P-Erk1/2 or P-Vav present in primary human T cells treated with rIL-37 (filled shapes) and activated with anti-CD3 antibody, and control cells not treated with rIL-37 (open shapes) activated with antibodies. Shown is the fold of P-Vav or P-Erk phosphorylation relative to actin. Statistical significance was determined using One-Way ANOVA with Tukey’s multiple comparisons test (a, b), or t-test (d). Data are the mean ± SEM of *n* = 3 biological replicates.

Having identified that *H.* *pylori* can utilize IL-37 to suppress immunity and augment colonization, we next examined the specific mechanisms whereby IL-37 impairs human immune cell functions and the development of *H.* *pylori*-specific immunity. To do this, we initially confirmed that recombinant IL-37 (rIL-37) could limit peripheral blood mononuclear cell (PBMC) IL-6 responses to LPS **(Supplementary Figure S9)** as we have previously reported.^12^ Having confirmed the effectiveness of rIL-37 in suppressing inflammatory responses, we next treated primary human immune cell subsets with rIL-37 and assessed how IL-37 altered their cellular functions. As *H.* *pylori* modulates T cell immunity as an immune evasion mechanism,^35^^,^^45^ we initially assessed the ability of rIL-37 to reduce the activation of peripheral blood human T and B cells. Treatment of T or B lymphocytes with rIL-37 reduced cell surface expression of the activation markers CD69 and CD86 following activation with anti-CD3 or anti-IgM antibodies, respectively (Figure 5a,b, *P* < 0.01). Furthermore, rIL-37 impaired T and B cell activation by reducing extracellular-signal-regulated protein kinase (Erk) Erk1/2 (*P* = 0.0472) and Vav (*P* = 0.001) kinase phosphorylation in primary human T cells (Figure 5c,d), and Erk phosphorylation in B cells **(Supplementary Figure S10)**. Collectively, these findings reveal that IL-37 functions to prevent human T and B cell activation by preventing the upregulation of surface activation markers and phosphorylation of Erk and Vav kinases, to ultimately limit the activation of adaptive immune cells in the host.

### IL-37 functions to limit human T cell migration and immune synapse formation between T and B cells to impair adaptive immune responses in the host

**Figure 6.**
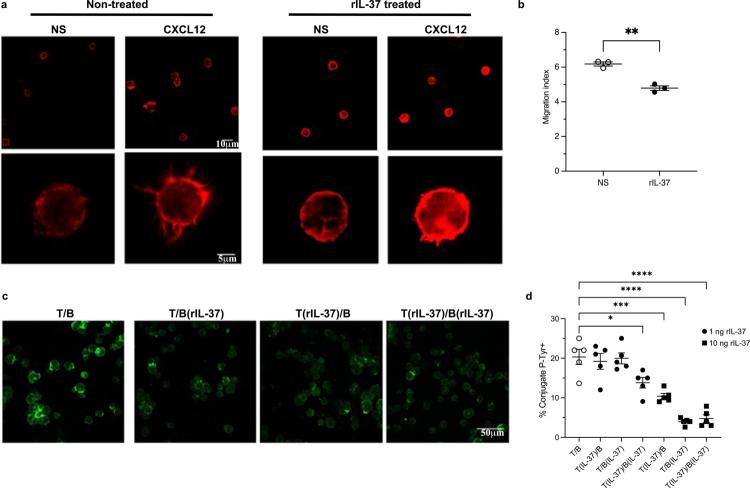
IL-37 functions to limit human T cell migration and immune synapse formation between T and B cells to impair adaptive immune responses in the host. (a) Jurkat T cells (1 × 10^5^/sample), either pre-treated with recombinant IL-37 (rIL-37, 10 ng/ml) or non-treated, were allowed to adhere to fibronectin coated microscope slides before stimulation for 5 min with 100 ng/ml CXCL12, or not stimulated (NS) as controls. Cells were stained using phalloidin and imaged by confocal microscopy. Shown are representative images from *n* = 3 independent experiments. (b) Quantification of the migration index of Jurkat T cells, treated with rIL-37 (rIL-37, filled shapes) or not treated as controls (NS, open shapes), towards a CXCL12 chemotactic gradient. Data are the mean ± SEM of *n* = 3 biological replicates. (c) Representative confocal microscopic images of immune synapse (IS) formation between primary human B cells (B) and primary human T cells (T). Cells were plated on poly-lysine-coated wells of diagnostic microscope slides and were allowed to adhere for 15 min and then fixed in 4% paraformaldehyde. Samples were incubated with an anti-phospho-tyrosine antibodies followed by labeling with an Alexa Fluor 488 labeled secondary antibody. Images shown are representative of IS formation between primary B and T cells not treated with rIL-37(T/B), primary T cells and B cells treated with IL-37 (T(rIL-37/B(rIL-37)), primary T cells pre-treated with IL-37 incubated with B cells (T(rIL-37)/B, or primary B cells treated with rIL-37 incubated with T cells ((T/B(rIL-37)). Images are representative of 3 independent experiments. Scale bar 50 µm. (d) Quantification of the percentage of conjugates with phospho-tyrosine polarization at the immune synapse (IS) between T cells and B cells from five donors, treated with varying amounts of rIL-37 (filled shapes) as indicated, or not treated as controls (T/B, open shapes). Shown is the percentage of phospho-tyrosine polarization of cells from each condition compared with non-treated cells. Data are the mean ± SEM of 5 biological replicates. Statistical significance was determined using (b) t-test, or (d) One-Way ANOVA with Dunnett’s multiple comparisons tests. *P < 0.05, **P < 0.01, ***P < 0.001, ****P < 0.0001.

As IL-37 was found to impair gastritis levels in response to *H.* *pylori* infection (Figure 4), in addition to preventing the activation of adaptive immune cells (Figure 5), we next examined the effect of IL-37 on T cell migration. To do this, we analyzed the effect of IL-37 on both T cell polarization and chemotaxis using a CXCL12 chemotactic gradient. We observed that the addition of IL-37 to Jurkat T cells prevented their polarization in addition to impairing their migration in response to a CXCL12 chemotactic gradient (Figure 6a,b, *P* < 0.01**)**, revealing that IL-37 restricted T cell activation and chemotaxis. The effect of rIL-37 on preventing chemotaxis was also confirmed using primary human T cells **(Supplementary Figure S11)**.

Upon activation, immune cells interact with each other to form an immune synapse, resulting in the initiation of an adaptive immune response. As IL-37 reduced immune cell recruitment, we next examined whether IL-37 also impaired immune synapse formation which can facilitate activation of effector immune cells. To do this, primary human T cells and B cells were each treated with rIL-37, or left untreated as controls, and incubated with each other in various combinations to determine if either cell type was affected in their ability to develop immune synapses. We observed that treatment of either primary human T or B cells with rIL-37 significantly reduced phospho-tyrosine immune synapse formation in a concentration-dependent manner (Figure 6c,d, *P* < 0.05, *P* < 0.001, *P* < 0.0001). Furthermore, the addition of IL-37 to both T and B cells further impacted immune synapse formation between T and B cells, revealing that IL-37 has a profound effect on preventing effective T and B cell responses. Taken together, these findings highlighting the ability of IL-37 to limit the activation, recruitment and induction of adaptive immune cell responses, and explains how IL-37 can prevent the induction of adaptive immunity.

### IL-37 impairs T and B cell proliferation and limits human gastric-derived *H.* *pylori*-specific T cell proliferation

**Figure 7.**
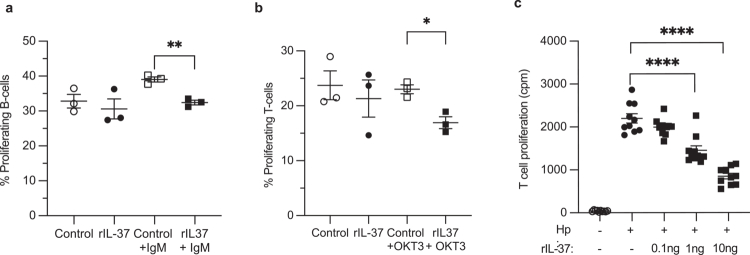
IL-37 impairs T and B cell proliferation and limits human gastric-derived *H.* *pylori* specific T cell proliferation. (a-b) Flow cytometric analysis of CFSE-labeled primary human (a) B and (b) T cells pre-treated with 10 ng/ml rIL-37 (rIL-37, filled shapes), or not treated as controls (open shapes), that were stimulated with anti-IgM or OKT3 antibodies (squares) for 72 h. Shown are the mean ± SEM of the percentage of CFSE low proliferating cells (% Proliferating cells) from 3 individuals. *P < 0.05, **P < 0.01 determined using Students T test. (c) Inhibition of proliferation of primary *H.* *pylori*-specific gastric T cells isolated from gastric biopsies obtained from 10 *H.* *pylori*-infected individuals. Gastric derived T cells were treated with rIL-37 (squares, 0.1−10 ng) or not treated with rIL-37 (circles), stimulated with *H.* *pylori* lysate (filled shapes), and proliferation in response to *H.* *pylori* stimulation was quantified. Data are the mean ± SEM of 10 biological replicates. Statistical significance was determined using One-Way ANOVA with multiple comparisons test. *P < 0.05, **P < 0.01, ****P < 0.0001.

Central to the mechanism whereby *H.* *pylori* modulates host immunity to promote lifelong colonization is the generation of an ineffective adaptive immune response that is incapable of clearing the infection. Having determined that IL-37 prevents the activation and immune synapse formation of T and B cells, we next examined the contribution of IL-37 to preventing the generation of effective T and B cell adaptive immune responses. To do this, CFSE-labeled primary human B and T cells were either treated or not with rIL-37 prior to their activation using anti-IgM or OKT3 antibodies respectively, and the percentage of proliferating T and B cells was quantified using flow cytometry. We found that IL-37 attenuated the proliferation of primary human T and B cells following antibody-mediated T cell receptor or B cell receptor activation (Figure 7a,b, *P* < 0.05, *P* < 0.01 respectively). Finally, as *H.* *pylori*-specific T cells are found in the gastric mucosa of *H.* *pylori*-infected individuals, we examined whether IL-37 could inhibit the proliferation of *H.* *pylori*-specific T cells located within the gastric tissue. To do this, *H.* *pylori*-specific T cells were isolated from the gastric mucosa of *H.* *pylori* infected individuals and were either treated with rIL-37 or not treated as controls, and their ability to proliferate in response to *H.* *pylori* antigen was determined. Impressively, rIL-37 substantially impaired proliferation of gastric-derived *H.* *pylori*-specific T cells in a dose-dependent manner, revealing that IL-37 can prevent the proliferation of T cells located within the gastric tissue of *H.* *pylori* infected individuals to further limit pathogen-specific immune responses (Figure 7c).

Collectively, these findings show that *H.* *pylori* induces the production of IL-37 by human gastric epithelial cells. IL-37 then functions to suppress innate and adaptive immune responses. Specifically, IL-37 regulates human B and T cell responses to *H.* *pylori* by inhibiting their cellular activation, immune synapse formation, chemotaxis and proliferative responses, thus causing pan-immunosuppression and contributing to chronic persistence.

## Discussion

*H.* *pylori* infects the gastric tissue of approximately half of the world’s population, causing a spectrum of diseases ranging from gastritis to gastric ulcers and cancer.^2^ Due to the ability of *H.* *pylori* to modulate host immunity, it can persist in the host for life.^1^ Some of the mechanisms used by *H pylori* to mediate tolerance involve modifications in key bacterial components in order to avoid immune detection by PRRs, in addition to harboring a range of virulence factors that function to suppress immune cell functions.^3^ For example, *H.* *pylori* can avoid detection by TLR4 and TLR5 due to modifications in LPS and flagellin, therefore preventing induction of PRRs and an inflammatory response.^7^^,^^46^ Furthermore, *H.* *pylori* can impair cellular immunity by limiting T cell and dendritic cell activation, proliferation and functions via a range of virulence factors, such as VacA, ADP heptose and γ-glutamyl transpeptidase,^8-11^^,^^47^^,^^48^ to ultimately promote gastric persistence and immune tolerance in the host.

In our study, we aimed to examine host immunomodulatory factors that may contribute to *H.* *pylori* mediated immunosuppression and persistence. To do this, we examined the role of IL-37 in modulating immunity to *H.* *pylori* and promoting persistence. Here we show that *H.* *pylori* exploits the powerful immunosuppressive and anti-inflammatory functions of IL-37 to impair host immunity and enhance colonization. These findings reveal that *H.* *pylori* harnesses IL-37 to abolish the development of effective innate, cellular and humoral immunity thereby maintaining colonization of the host.

We identified that IL-37 expression was significantly increased within epithelial cells in the gastric tissues of *H.* *pylori* infected individuals, and that *H.* *pylori* induced the secretion of IL-37 by human gastric epithelial cells and gastric mucosoids. Intracellular storage of IL-37 at steady-state and the secretion of cleaved IL-37 upon stimulation with *H.* *pylori*, i.e. IL-37 exhibiting alarmin-like properties, is consistent with our earlier report identifying that intracellularly stored IL-37 is secreted by blood cells upon PRR-mediated activation.^31^ These findings reveal that IL-37 acts as an anti-inflammatory alarmin not only in blood, but also in epithelial cells, and in response to *H.* *pylori* infection.

As IL-37 is produced by host cells in response to PRR activation,^12^ we next examined the PRRs responsible for *H.* *pylori* mediated IL-37 secretion by gastric epithelial cells. Gastric epithelial cells express TLR2, TLR4 and NOD1, and their expression is increased during *H.* *pylori* infection.^49^^,^^50^ As *H.* *pylori* can activate TLR2, TLR4 and NOD1,^17^^,^^41^^,^^51^^,^^52^ the contribution of these PRRs to *H.* *pylori* mediated IL-37 production by epithelial cells was determined. *H.* *pylori* induced the production of IL-37 by epithelial cells in a TLR2, TLR4 and NOD1-dependent manner, thus suppressing further activation of these PRRs. Therefore, these findings provide evidence as to how *H.* *pylori* can impair PRR activation in epithelial cells to ultimately limit PRR-mediated responses. As *H.* *pylori* LPS is atypically detected via TLR2,^7^ and *H.* *pylori* HP0175 can activate TLR4 in AGS cells,^53^ future studies should focus on the contribution of these bacterial components to IL-37 induction and IL-37-mediated immunomodulation in the host. Moreover, our findings identify the previously unknown contribution of NOD1 to the production of IL-37, in addition to CagA being additive to IL-37 production, and propose yet another mechanism whereby *H.* *pylori* cagPAI positive strains which are often attributed to more severe disease^2^ can further modulate host immunity to promote pathogenesis.

Furthermore, we identified that IL-37 expression by epithelial cells impaired TLR2, TLR4 and NOD1 activation by *H.* *pylori*, in addition to TLR2 and TLR4-mediated IL-8 production in response to *H.* *pylori* stimulation. These findings propose yet another pathway whereby *H.* *pylori* can prevent cytokine production and thereby limit immune cell recruitment in response to a chemotactic gradient within the gastric tissue, to ultimately enhance colonization and promote the development of a slow and chronic gastric inflammatory disease state. This hypothesis is further supported by our *in vivo* data, which revealed that human IL-37 reduced acute and chronic gastritis levels in *H.* *pylori* infected animals compared to control *H.* *pylori* infected mice that lacked IL-37. Moreover, *H.* *pylori* infected IL-37tg mice exhibited enhanced colonization and reduced *H.* *pylori*-specific antibody responses compared to infected wild type mice, revealing that IL-37 functioned to enhance gastric colonization in addition to promoting dysregulation of host immunity. These findings not only provide an explanation as to how *H.* *pylori* can function to suppress host immune responses, but also suggest that IL-37 may be contributing to the slow progression of chronic gastritis in infected individuals.

Examination of the effect of IL-37 on various immune cell subsets revealed the multi-pronged approach used by IL-37 to suppress T and B cell functions. We found that IL-37 prevented the activation of T and B cells and their recruitment towards a chemotactic gradient, suggesting that *H.* *pylori* mediated IL-37 production by epithelial cells functions to impair the activation and recruitment of immune cells to the gastric tissue. Moreover, IL-37 prevented the development of immune cell synapses between T and B cells, highlighting further that IL-37 can result in ineffective T and B cell responses to *H.* *pylori*. Finally, using primary human T and B cells, and *H.* *pylori* specific gastric derived human T cells, we showed that IL-37 functioned to abolish their proliferative response. This provides further explanation as to the ineffectiveness of T and B cells within the gastric mucosa and the mechanism whereby *H.* *pylori* can prevent the development of an effective *H.* *pylori*-specific T cell response required to clear the infection.

Collectively, these findings provide the first explanation as to how *H.* *pylori* can mediate broad anti-inflammation and pan-immunosuppression in the host, by using a multi-faceted approach to prevent innate immune receptor activation, immune cell recruitment and ultimately impairing immune cell functions that are all mediated via the production of IL-37 by the host in response to *H.* *pylori* infection. These findings reveal that *H.* *pylori* induces the production and secretion of IL-37 by gastric epithelial cells in a PRR and cagPAI dependent manner. IL-37 then functions to enhance colonization while limiting gastritis and antibody responses, in addition to preventing cellular activation and migration, and the induction of *H.* *pylori*-specific adaptive immunity. Furthermore, we provide the mechanistic explanations for IL-37 mediated attenuation of pathogen-specific human T and B cell responses. Taken together, these findings provide novel insights into how *H.* *pylori* manipulates the host into producing and secreting IL-37 to impair host immunity and promote its persistence. Moreover, we propose that this broad IL-37-mediated immunosuppression promotes the host’s inability to eradicate *H.* *pylori*. As *H.* *pylori* causes massive morbidity and mortality globally, and with antimicrobial resistance increasing and no vaccine available, the magnitude of *H.* *pylori* infections and the prevalence of gastric cancer is predicted to rise.^2^ Therefore, the unmet need for novel therapeutic interventions and targets to combat *H.* *pylori* infection is highly urgent. Our work represents a critical advance in the understanding of *H.* *pylori*-mediated disease and identifies gastric IL-37 as a therapeutic target with enormous potential to combat *H.* *pylori* infection and *H.* *pylori*-mediated diseases, such as gastric cancer.

## Supplementary Material

Pathirana sup methods with sup data figures.docx Pathirana sup methods with sup data figures.docx

Resubmission Pathirana Supplementary material.pdf Resubmission Pathirana Supplementary material.pdf

## Data Availability

The data supporting the findings of this study are available within the article and in the supplemental material. All proteomic data have been deposited in PeptideAtlas Consortium via the PeptideAtlas proteomics repository: PASS01395. http://www.peptideatlas.org/PASS/PASS01395.
