# Supplementary material for: Helicobacter pylori induces the production of interleukin-37 to promote broad immunosuppression and enhance colonization
Source: Gut Microbes. 2026 Feb 14;18(1):2618860. doi: 10.1080/19490976.2026.2618860 (PMC12915765; doi:10.1080/19490976.2026.2618860)
Supplement: Pathirana sup methods with sup data figures.docx [file KGMI_A_2618860_SM1424.docx]

**Supplementary Material**

**TITLE: *Helicobacter pylori* induces the production of interleukin-37 to promote broad immunosuppression and enhance colonization.**

**Supplementary Methods:**

**AGS cell viability in response to *H. pylori* infection.**

Viability of *H. pylori* -stimulated AGS cells was determined using the CellTiter-Glo® 2.0. Cell Viability assay (Promega). In brief, AGS cells were seeded in opaque-walled 96-well cell culture plates (2×10^4^ cells/well) in 100μl of complete culture medium and grown overnight at 37°C in 5% CO_2_. The following day, complete culture medium was replaced with plain RPMI-1640 culture medium without antibiotics and AGS cells were stimulated for 1 h with *H. pylori* at the MOI indicated (MOI 1, 10, 100 and 1000). The media was then replaced with plain RPMI-1640 without antibiotics, and AGS cells were incubated for an additional 23 h. Cell viability was then determined according to the manufacturer’s instructions, with 100μl of medium-containing cells being added to an equivalent volume of CellTiter-Glo® 2.0 Reagent. The contents were then mixed for 2 mins with shaking at 200 rpm, the plate was incubated at room temperature for 10 mins to stabilise the luminescent signal and luminescence was measured using a plate reader (CLARIOstar, BMG Labtech).

**Generation of gastric human mucosoids and co-culture assays to examine IL-37 production.**

Human gastric mucosoids were obtained from gastric mucosa samples obtained from three healthy subjects (age: 45, 52, 54 years), negative for *H. pylori* infection, enrolled in the study after their informed consent under the approval of the Florence Ethics Committee 322 code: BIO 114.013_AOUC.

Briefly, gastric primary cells derived from the disaggregation of freshly isolated gastric tissue glands as previously (1) were seeded 4x10^5^ cell/200µl of medium and cultivated on collagen-coated (TeloCol^®^-6 bovine collagen solution, Advanced Biomatrix) porous transwell inserts with 1 μm pore polycarbonate membranes in a 12 well plate (ThinCert™, Greiner bio-one), at the air-liquid interface (ALI). Mucosoid seeding medium was also added in each well of the plate containing a filter, which was comprised of Advanced DMEM/F12 with NEAA, Na-pyruvate, and without L-glutamine (Gibco, Thermo Fisher Scientific) supplemented with 25 % (v/v) R-Spondin conditioned medium, 25 ng/ml Wnt Surrogate-Fc Fusion Protein (N001, U-Protein Express B.V.), 2 % (v/v) B-27 Supplement (50×) (17504–044, Thermo Fisher Scientific), 10 mM Nicotinamide (N0636- 100G, Sigma Aldrich), 1 % (v/v) Penicillin-Streptomycin (100×) (15140–122, Thermo Fisher Scientific), 1 % (v/v) N-2 Supplement (100×) (17502–048, Thermo Fisher Scientific), 20 ng/ml Human EGF (PHG0311, Thermo Fisher Scientific), 1 μM TGF-β RI Kinase Inhibitor IV (Alk-I) (616454, Calbiochem), 150 ng/ml Human FGF-10 (100-26, PeproTech), 150 ng/ml Human Noggin (120-10C, PeproTech), 10 nM Human [Leu 15 ]-Gastrin I (G9145, Sigma-Aldrich) and 7.5 μM ROCK inhibitor (Y-27632) (Y0503, Sigma Aldrich).

Cells were incubated at 37°C with 5 % CO_2_ for 4 days. On day 3, the medium overlying the cells was removed in order to start the ALI culture. The medium in the bottom chamber was replaced with mucosoid seeding medium that was additionally supplemented with 1.5 μM ROCK inhibitor (Sigma Aldrich) twice a week and the mucous accumulated inside the filter was removed at the same time. Gastric mucosoids were grown using this method for 12-18 days to generate a cell layer with complete barrier integrity; then they could be harvested, resuspended and reseeded.

For each healthy subject, on the day of the experiment, 27 derived gastric mucosoids were washed using PBS and passed into a new plate containing porous transwell inserts (1 μm pore polycarbonate membranes), filled with 400 µl of culture medium/well (without antibiotics). Mucosoids (3 per condition) were incubated with either PBS or with a single *H. pylori* strain at an MOI of 100: either 251, 251 Δ*cag*PAI, 251 Δ*cag*A, MMC2, MMC6, MMC10, MMC17 or MMC19, and all samples were tested in triplicate. Gastric mucosoids were incubated for 36 hours at 37°C with 5 % CO_2_ atmosphere, after which the medium inside the filter and in the bottom tank was harvested, centrifuged IL-37 levels quantified by immunoassay Luminex technology (IL-37 Human ProcartaPlex™ Simplex Kit, Invitrogen Thermo Fisher) as per the manufacturer’s protocol. In brief, capture beads, samples (50 µl) and IL-37 standard dilutions (detection range 4.22 - 17300 pg/ml) were added to the 96 well plate supplied with the kit. After 120 minutes of incubation at RT, the plate was washed, and detection antibody was added 30 minutes. The plate was then washed, streptavidin-PE was added for 30 minutes, and the plate was washed again. Reading buffer was then added to the plate and IL-37 levels were quantified using the Luminex 200 System, and data were acquired using Luminex xPONENT software. IL-37 concentrations values falling below the lower limit of quantification (LLOQ) of the Luminex assay were replaced with one-half the respective LLOQ for descriptive purposes.

**Supplementary References**

1. Schlaermann P, Toelle B, Berger H, Schmidt SC, Glanemann M, Ordemann J, et al. A novel human gastric primary cell culture system for modelling *Helicobacter pylori* infection in vitro. Gut. 2016;65(2):202-13.

**Supplementary Figure S1. *H. pylori* induces IL-37 secretion by AGS cells.**

AGS cells were stimulated with *H. pylori* 251 at an increasing MOI (MOI 1, 10 100 or 1000, filled circles), not stimulated as controls (NS, open circles). IL‑37 in supernatants was detected by immunoblotting 24 h post stimulation. Fold-change in secreted IL‑37 relative to *H. pylori* 251 stimulated cells (MOI 100) is depicted. Symbols are individual data points of 3 biological replicates, with the mean ± SEM shown. Statistical significance determined by One-way ANOVA with Tukey’s multiple comparison test.

* P < 0.05, ** P < 0.01.

**Supplementary Figure S2. AGS viability post infection with *H. pylori* strains at an increasing MOI.**

AGS cells were stimulated with either *H. pylori* 251 (251), *H. pylori* ΔcagPAI (cagPAI), or *H. pylori* isolates MMC2, MMC6, MMC10, MMC17 or MMC19 at an MOI of either 1, 10, 1000 or 1000 for 1 h (filled circles) or media alone as a control (open circles, Control). Media was them removed and replaced with plain RPMI media and cells were further incubated for a total of 24 hours.

Cell viability was then determined using the Cell Titer Glow assay, and percentage viability was determined compared to PBS stimulated control cells. Symbols are individual data points of 3 biological replicates, with the mean ± SEM shown.

Statistical significance determined by Two-way ANOVA with Dunnett’s multiple comparison test relative to media alone control cells (Control).

*P < 0.05, * * P < 0.01, *** P < 0.001.

| **Identified Sequence** | **Mass** | **Unique (Proteins)** | **PEP** |
| --- | --- | --- | --- |
| ^127^GQSHPSLQLK^136^ | 1093.588 | yes | 0.011369 |
| ^106^GSPILLGVSK^115^ | 969.5859 | yes | 0.011011 |
| ^198^HIEFSFQPVCK^208^ | 1390.67 | yes | 5.06E-05 |
| ^59^KFSIHDQDHK^68^ | 1253.615 | yes | 0.099283 |
| ^152^RPFIFYR^158^ | 997.5498 | yes | 0.012026 |
| ^69^VLVLDSGNLIAVPDK^83^ | 1551.887 | yes | 0.000 |

**Supplementary Table S1. Detection of IL-37 protein in the supernatants of *H. pylori* stimulated AGS cells.**

IL‑37 proteome analysis from *H. pylori* 251 stimulated AGS cell supernatants (cleaved IL‑37 protein). Quantitative proteomic profiling revealed identification of IL‑37 (human). Peptide sequence of IL‑37 protein (UniProtKB ID IL37_HUMAN) identified in AGS cell supernatants using mass spectrometry. Six peptides spanning between amino acid 59-68, 69-83, 106-115, 127-136, 152-158, and 198-208 unique in IL‑37 were identified. PEP = posterior error probabilities. n=3 combined analyses.

**
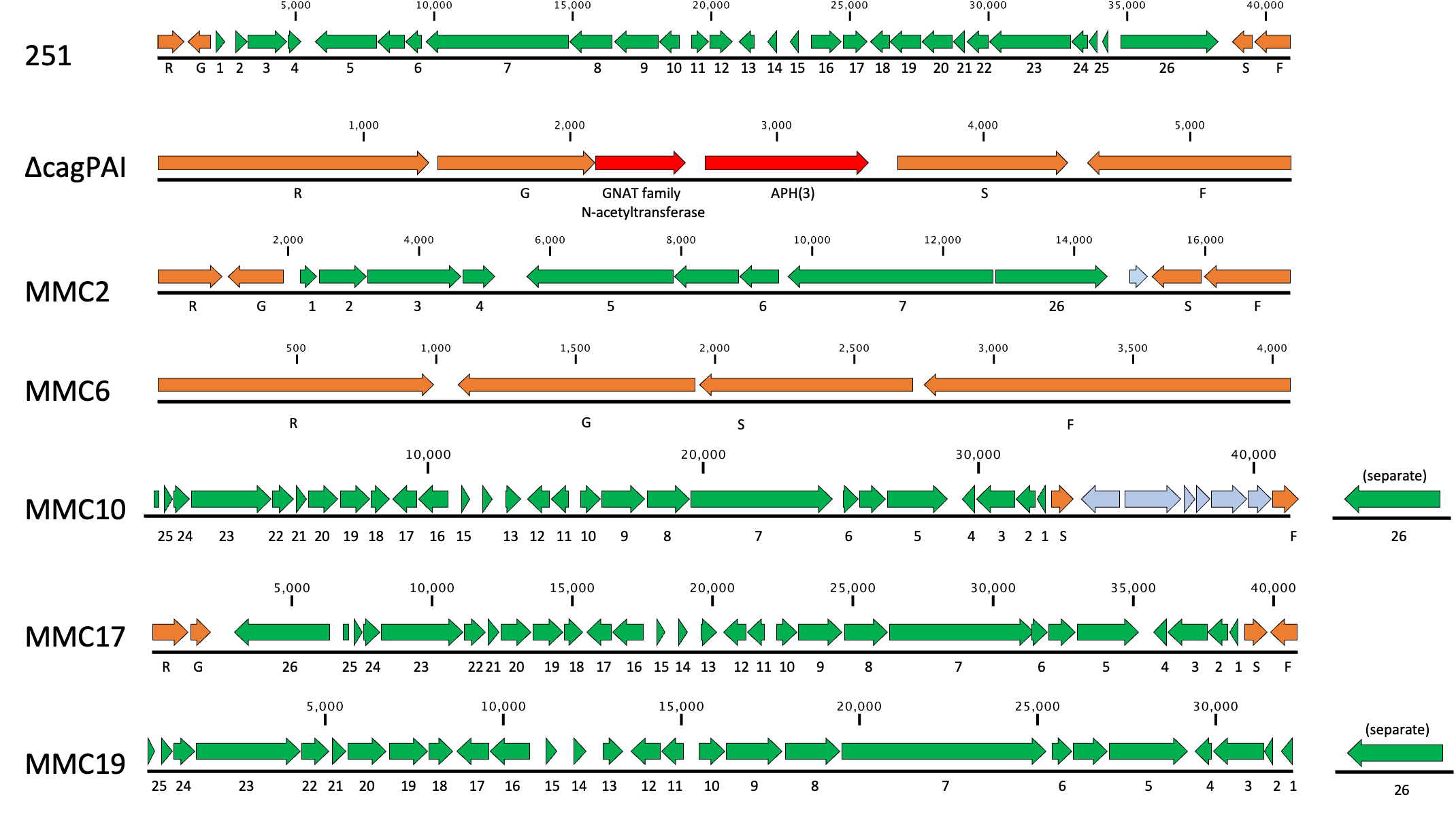
**

**Supplementary Figure S3. Annotated cagPAI region of *H. pylori* isolates.**

Genomic DNA obtained from *H. pylori* 251, 251cagPAI isogenic mutant (ΔcagPAI), and *H. pylori* clinical isolates MMC2, MMC6, MMC10, MMC17 and MMC19 was sequenced and assembled. Shown is the annotated cagPAI region of each strain. Numbered genes (shaded in green) indicate cag gene identity. Other gene annotations (shaded in orange) include; R (rho transcriptional terminator); G (Glutamate racemase); S (Sel1-like repeat region); F (Flagellin deglycosylase). Genes of unknown function are shaded in blue and unannotated. The cagPAI region is partially present in MMC2 (deletion between cag7-cag26 is evident) and absent in MMC6 but intact in the remainder of the samples. Cag26 assembled into separate contigs in MMC10 and MMC19.

**Supplementary Figure S4. Human gastric mucosoids secrete IL-37 in response to *H. pylori* infection.**

Human gastric mucosoids generated from gastric tissue obtained from three healthy humans were infected with either *H. pylori* 251, *H. pylori* 251 mutants (CagPAI, CagA), *H. pylori* clinical isolate strains (MMC-2,6,10,17,19), or PBS as a control (open circles) for 36 hours. IL‑37 secreted into cell culture supernatants was quantified using an IL‑37 Luminex assay. Shown are data obtained from n=3 biological replicates, with the mean ± SEM shown. Statistical significance determined by One-way ANOVA with Dunnett’s multiple comparison test, with all samples compared to PBS controls. **** *P* < 0.0001.

**Supplementary Figure S5. *H. pylori* cagA contributes to but is not essential for IL‑37 secretion by AGS cells.**

**(a)** AGS cells were stimulated with either *H. pylori* 251 (251, MOI 10 or MOI 100), *H. pylori* 251 cagPAI mutant (cagPAI, MOI 10 or MOI 100), 251 cagA mutant (cagA, MOI 10 or MOI 100), or not stimulated as controls (NS). IL‑37 in supernatants was detected by immunoblotting 24 h post stimulation. Cleaved IL‑37 is indicated. Blot is representative of n=4 biological replicates.

**(b)** Densitometry of IL‑37 secreted by AGS cells in response stimulation with either *H. pylori* 251 (251, MOI 100), *H. pylori* 251 cagPAI (cagPAI, MOI 100,) *H. pylori* 251 cagA (cagA, MOI 100,) or not stimulated as controls (NS, open circles). Fold-change in secreted IL‑37 relative to *H. pylori* 251 stimulated cells (MOI 100) is depicted. Symbols are individual data points of 4 biological replicates, with the mean ± SEM shown. Statistical significance determined by One-way ANOVA with Tukey’s multiple comparison test. **** P < 0.0001.

**Supplementary Figure S6. Gastric epithelial (AGS) cell viability.**

AGS cells pre-treated for 1 h with either the NOD1 inhibitor ML130 (5 μM), NLRP3 inhibitor MCC950 (50 μM), pan-caspase inhibitor Z-VAD-FMK (20 μM), caspase 1 inhibitor Z-WHED-FMK (20 μM), DMSO (as vehicle) or non-treated cells were stimulated with *H. pylor*i 251 at an MOI of 100 for 1 h. Following stimulation, AGS cells were incubated for a total of 24 h in serum-free media in the presence of either ML130 (5 μM), MCC950 (50 μM), Z-VAD-FMK (20 μM), Z-WHED-FMK (20 μM), DMSO or without any inhibitor (Control) and the percentage of cell cytotoxicity was determined by using a MTT (3-(4,5-dimethylthiazol-2-yl)-2,5-diphenyltetrazolium bromide) assay according to manufacturer’s instructions (Abcam). The % cytotoxicity in each of the groups, compared to control AGS cells, from three biological replicates are presented, with the mean and SEM shown. There was no statistical difference in the viability of AGS cells between each group, compared to control cells, as determined by One-Way-ANOVA with Dunnett’s multiple comparison test.

**Supplementary Figure S7. IL-37 suppresses IL-8 responses in TLR4-expressing AGS cells.**

AGS cells were transiently transfected for 24 h with an IL‑37 expression construct (IL‑37, triangles and diamonds), or a control construct (Control plasmid, circles). Cells were also co-transfected with a TLR4‑MD2 expression construct (TLR4) as indicated. The following day, AGS cells were stimulated with *E. coli* LPS (100ng/ml) for 16 hr and IL‑8 secreted into the culture supernatants was quantified by ELISA. Shown are 3 replicates with the mean ± SEM. Data are representative of 3 replicate experiments. Statistical significance determined by One-way ANOVA with Tukey’s multiple comparison test. ***P* < 0.01, **** *P* < 0.0001.

**Supplementary Figure S8. *H. pylori* SS1 induces IL-37 secretion by AGS cells.**

**(a)** AGS cells were stimulated with either *H. pylori* 251 (251, MOI 10 or MOI 100), *H. pylori* SS1 (SS1, MOI 10 or MOI 100), or not stimulated as controls (NS). IL‑37 in supernatants was detected by immunoblotting 24 h post stimulation. Cleaved IL‑37 is indicated. Blot is representative of n=3 biological replicates.

**(b)** Densitometry of IL-37 secreted by AGS cells in response stimulation with either *H. pylori* 251 (251, MOI 100, filled circles), *H. pylori* SS1 (SS1, MOI 100, filled circles) or not stimulated as controls (NS, open circles). Fold-change in secreted IL‑37 relative to *H. pylori* 251 stimulated cells (MOI 100) is depicted. Symbols are individual data points of 3 biological replicates, with the mean ± SEM shown. Statistical significance determined by One-way ANOVA with Tukey’s multiple comparison test.

** P < 0.01, *** P < 0.001. ns= not significant.

**Supplementary Figure S9. Confirming the immunosuppressive effects of rIL-37 on IL-6 PBMC responses to LPS.**

PBMCs obtained from 10 individuals were stimulated with 10 ng of recombinant IL‑37 (rIL‑37) in the presence or absence of LPS (1 mg/ml). IL‑6 levels secreted in the culture supernatant were quantified by ELISA. Shown are the mean ± SEM of IL‑6 responses of PBMCs obtained from 10 individuals. **** *P*< 0.0001 calculated using one-way ANOVA with Tukey multiple comparisons test.

**Supplementary Figure S10. Recombinant IL-37 impairs ERK phosphorylation in human B cells.**

Human B cells purified from peripheral blood of healthy volunteers were pre-treated with recombinant IL‑37 (rIL‑37, 10 ng/ml) for 24 h, or not treated as controls, followed by activation with antagonistic anti-IgM antibody.  **(a)** The cells were lysed and Erk1 phosphorylation was detected by Western blot analysis using rabbit polyclonal antibodies against Erk1/2 (T202/204). Detection of actin was used as a loading control. Data shown are representative of two independent experiments. **(b)** Relative densitometry values of Erk1 phosphorylation (P-Erk) from non-treated or rIL‑37 treated human B cells. Data are shown as fold change compared with basal expression levels relative to actin. Shown are data obtained from n=2 biological replicates, with the mean ± SEM shown.

**Supplementary Figure S11. IL-37 may impair primary T cell migration.**

Quantification of the migration index of human primary T cells, treated with IL‑37 (rIL‑37, filled shapes) or not treated as controls (NS, open shapes), towards a CXCL12 chemotactic gradient. Data are the mean ± standard error of the mean (SEM) of n=3 biological replicates.
